# Trajectories of Childhood Adversity and Eating Disorders in Adolescence

**DOI:** 10.1002/eat.24514

**Published:** 2025-07-28

**Authors:** Andrea Joensen, Leonie K. Elsenburg, Else Marie Olsen, Claus Thorn Ekstrøm, Naja Hulvej Rod, Katrine Strandberg‐Larsen

**Affiliations:** ^1^ Section of Epidemiology, Department of Public Health University of Copenhagen Copenhagen Denmark; ^2^ Copenhagen Health Complexity Center, Department of Public Health University of Copenhagen Copenhagen Denmark; ^3^ Outpatient Clinic for Eating Disorders Psychiatric Centre Ballerup Ballerup Denmark; ^4^ Section of Biostatistics, Department of Public Health University of Copenhagen Copenhagen Denmark

**Keywords:** diagnostic disparities, eating disorders, trajectories of childhood adversities

## Abstract

**Objective:**

Childhood adversities are linked to eating disorders (EDs), but their cumulative and evolving nature is often overlooked. This study employs a comprehensive measure of adversities, captured through trajectories across ages 0–9, to examine associations with (1) clinically diagnosed EDs at ages 10–18 and (2) a composite outcome of diagnosed and self‐reported ED symptoms at age 18. Differences between diagnosed and non‐diagnosed EDs were also assessed.

**Methods:**

Data from children born in Denmark between 1996 and 2003 (total population; *N* = 500,240) and participants in the 18‐year follow‐up of the Danish National Birth Cohort (DNBC‐18; *N* = 43,687, 50% of those eligible for invitation) were used. Diagnosed EDs were identified through national registers, and non‐diagnosed EDs through the DNBC‐18. Adversities were grouped into three predefined dimensions: material deprivation, loss or threat of loss, and family dynamics. A group‐based multi‐trajectory model identified trajectories from ages 0–9. Associations were analyzed using quasi‐Poisson and multinomial logistic regression.

**Results:**

Four trajectory groups were identified: low adversity (72.1%), material deprivation (18.0%), loss or threat of loss (5.5%), and high adversity (4.4%). < 1% had a diagnosed ED, while 2.5% had a non‐diagnosed ED, both more common in females. While no overall associations were found in the total population, the high adversity group had a higher risk of eating disorders not otherwise specified (EDNOS) compared to the low adversity group (IRR = 1.58, 95% CI: 1.16–2.12). In the DNBC‐18 analyses, a higher risk in the high adversity group compared to the low adversity group, was entirely driven by non‐diagnosed EDs (RRR = 1.73, 95% CI: 1.37–2.20).

**Conclusion:**

Individuals exposed to high adversity had a higher risk of EDs, particularly non‐diagnosed cases, highlighting a diagnostic gap shaped by differences in symptom recognition, help‐seeking, and access to care.

**Summary**

- Childhood adversities are associated with eating disorders (EDs), but most research has examined individual adversities rather than their cumulative effects.
- This study employes a trajectory‐based approach, using 12 childhood adversity measures from national registers, grouped into three predefined dimensions: material deprivation, loss or threat of loss, and family dynamics to identify four distinct adversity patterns from ages 0–9.
- Childhood adversities were not associated with overall diagnosed EDs, but the high adversity group had a higher risk of diagnosed EDNOS compared to the low adversity group. When including both diagnosed and non‐diagnosed EDs, high adversity was associated with any EDs, entirely driven by non‐diagnosed ED cases.
- Findings highlight a diagnostic gap, likely shaped by differences in treatment‐seeking, symptom recognition, and access to care, reinforcing the need to reduce referral barriers for vulnerable children and adolescents.

## 1 Introduction

Eating disorders (EDs) are severe psychiatric illnesses associated with high psychiatric co‐morbidity and risk of premature death (Larsen et al. 2024). They are more prevalent among females, with half of all diagnoses occurring during adolescence (Beck et al. 2024; Larsen et al. 2024). EDs are often marked by a prolonged and frequent chronic course, with a concerningly low proportion of affected individuals seeking or receiving treatment (Forrest et al. 2017). It is estimated that only one in three individuals with an ED seek treatment, with males having an even lower probability of seeking treatment (Ali et al. 2017; Bohrer et al. 2017; Forrest et al. 2017; Mitchison and Hay 2014; Swanson and Field 2016). Among adolescents, the treatment‐seeking rate is estimated at approximately 20% (Forrest et al. 2017).

Previous prospective studies suggest that childhood adversities such as parental psychiatric and somatic illness, family disruption, and familial death may contribute to the risk of EDs (Larsen et al. 2017; Su et al. 2016). In these studies, adversities were typically operationalized as binary indicators of presence or absence, without accounting for their timing, duration, or cumulative exposure. A Danish register‐based study examining nine specific childhood adversities during the first 6 years of life found that individuals exposed to these adversities had a higher risk of diagnosed bulimia nervosa (BN) and eating disorders not otherwise specified (EDNOS), but a lower risk of diagnosed anorexia nervosa (AN) during follow‐up to age 24 (Larsen et al. 2017). Another register‐based study using data from both Danish and Swedish registers found a higher risk of diagnosed EDs, primarily driven by BN and mixed ED presentations (e.g., overeating associated with other psychological disturbances), but not AN among those exposed to early life stress following the death of a close relative (Su et al. 2016). A systematic review of childhood maltreatment, including sexual abuse, physical violence, and other adversities, found a higher risk of AN among those exposed to adversities. However, this study did not distinguish variations in study design, adversity severity, or whether AN was clinically diagnosed or self‐reported (Amiri and Sabzehparvar 2024).

Register‐based studies are limited to clinically diagnosed cases from treatment settings (Swanson and Field 2016) and are likely to suffer from bias as those with treatment represent a selected subset of all individuals suffering from EDs (Ali et al. 2017). Several population‐based studies, defined as research including individuals not necessarily seeking medical treatment, have consistently found higher risks of self‐reported ED symptoms associated with self‐reported childhood adversity (Bodell et al. 2011; Copeland et al. 2015; Loth et al. 2008; Thomas et al. 2020). However, the definition of ED symptoms varies widely across studies, with only one aligning its outcome definition with clinical criteria (DSM‐IV) for AN, BN, or binge eating disorder (BED) (Johnson et al. 2002).

While previous studies have mainly focused on single adversities experienced, this study aims to investigate the trajectories of childhood adversities, capturing their timing, duration, and cumulative patterns experienced during the first 9 years of life. This approach provides a deeper understanding of how the cumulative nature of adversities is associated with the risk of EDs during adolescence. We used a large population‐based sample with longitudinal registry and self‐reported data to examine the association between trajectories of childhood adversities and EDs. This included both clinically diagnosed EDs and non‐diagnosed EDs, the latter reflecting threshold‐level manifestations of clinical EDs that have not been formally diagnosed.

The aim of this study was twofold. First, we examined the association between trajectories of childhood adversity experienced during ages 0–9 and the onset of diagnosed EDs between the ages 10–18 among all children born in Denmark from 1996 to 2003 (*N* = 500,240). Second, we examined the association between trajectories of childhood adversity and any EDs, including diagnosed EDs between ages 10–18 and self‐reported ED symptoms at age 18 (*N* = 43,687). We further assessed whether the associations varied for diagnosed and non‐diagnosed EDs. We hypothesized that individuals exposed to higher levels of childhood adversity would have a higher risk of non‐diagnosed EDs, reflecting barriers to treatment and diagnosis.

## 2 Methods

The preregistered study protocol is available on the Open Science Framework (Joensen 2024).

### 2.1 Design and Study Population

For the first aim, we included all children born in Denmark from 1996 to 2003, identified through national registers, including the Danish Medical Birth Register, constituting the total population for this study. For the second aim, we utilized data from the Danish National Birth Cohort (DNBC), a cohort nested within the general population, comprising 96,726 children born in Denmark during the same period, with longitudinal cohort data collected from prenatal life through early adulthood (Strandberg‐Larsen et al. 2025). Between 2016 and 2021, participants born into the DNBC were invited to complete an online survey (DNBC‐18) upon turning 18 and 3 months, providing information on ED symptoms. The background population for this study included all live‐born children whose mothers were eligible for recruitment into the DNBC. Recruitment for the DNBC began as a pilot project in one county in 1996 and was gradually expanded nationwide by 1999. This population represents a subset of the total population born in Denmark from 1996 to 2003 and was used to construct sample weights for analyses involving the DNBC‐18 subsample (used in the study's second aim). In Denmark, individuals are assigned a unique personal ID number, enabling individual‐level linkage to various administrative registers (Pedersen 2011). This facilitated the linkage of DNBC data with information on population characteristics, childhood adversities, and ED diagnosis.

Specific exclusions were applied to the different populations. In both populations, we excluded children born outside Denmark due to missing information on childhood adversities before immigration. Additionally, children who could not be linked to a parent in the register were excluded. Children who emigrated, died, were diagnosed with an ED before age 10, or had missing covariate data were also excluded. In the DNBC‐18, participants with missing data on any items related to ED symptoms were also excluded.

### 2.2 Measures of Childhood Adversities

We utilized longitudinal data from Danish national registries on 12 childhood adversities and their timing, as used previously in the DANLIFE cohort study (Bengtsson et al. 2019; Rod et al. 2020). All adversities occurring between 0 and 9 years of the child's life were counted per year of life. A few adaptations were made to the adversity definitions outlined in the DANLIFE cohort. A detailed overview of the definitions and adaptations is presented in Table S1. A panel of experts in stress, child health, and child psychology identified three predefined dimensions: material deprivation (i.e., family poverty and parental long‐term unemployment), loss or threat of loss within the family (i.e., parental severe somatic illness, sibling severe somatic illness, and death of a parent or sibling) and family dynamics (i.e., parental separation, being placed in foster care, parental psychiatric illness, sibling psychiatric illness, and parental alcohol or drug abuse) (Rod et al. 2020).

### 2.3 Measures of EDs

#### 2.3.1 Diagnosed EDs

Information regarding the diagnosis of EDs was retrieved by linkage to the Danish National Patient Register (NPR) including all hospital contacts from both private and public hospitals, inpatient as well as outpatient (Lynge et al. 2011) and the Danish Psychiatric Central Research Register (PCRR) (Mors et al. 2011). Both primary and secondary ICD‐10 diagnoses of AN (F50.0, F50.1), BN (F50.2, F50.3) and EDNOS (F50.8, F50.9) were included. As binge eating disorder (BED) is not formally classified in ICD‐10, it could not be captured through register‐based diagnoses. The onset date was defined as the admission date of the first hospital contact with one of the above diagnoses from ages 10–18, and overall diagnosed ED was defined as any of the above ED subtypes given at first contact.

#### 2.3.2 Self‐Reported Eating Disorder Symptoms

The DNBC‐18 included items adapted from the McKnight Risk Factor Survey, including weight and shape concerns (Field et al. 2001; Shisslak et al. 1999) and the Youth Risk Behavior Surveillance System survey, including binge eating, self‐induced vomiting, and using laxatives (Brener et al. 2013; Kann et al. 2018). All questions refer to behaviors and concerns over the past year. These items have been validated as instruments to screen adolescents for binge eating and purging behaviors (Field et al. 2004). The total number of items varied from 15 to 20 items, depending on positive or negative responses. These items were used to define threshold measures of AN, BN, and BED according to DSM‐5 symptom domains and inspired by definitions used in previous studies (Micali et al. 2015; Stice et al. 2017). Body mass index (BMI) was defined as weight in kilograms divided by height in meters squared (kg/m^2^), based on self‐reported height and weight assessed in the DNBC‐18. A detailed overview of self‐reported ED symptom definitions is presented in Table 1. Individuals with symptoms fulfilling any of these definitions were categorized as self‐reported ED cases.

**TABLE 1:** Self‐reported ED symptoms definitions in the DNBC‐18.

AN	BMI < 18.5 AND (high weight/shape concern OR at least monthly fasting OR engaged in excessive exercise)
BN	BMI ≥ 18.5 AND weekly binge eating AND (weekly purging^a^ OR engaged in excessive exercise)
BED	Weekly binge eating AND eating until stomach hurt or felt sick AND feeling guilt after binge eating AND absence of purging^a^ AND fasting AND excessive exercise

^a^ Purging includes both self‐induced vomiting and/or the use of laxatives.

#### 2.3.3 Any EDs: Combining Diagnosed and Self‐Reported Eating Disorder Symptoms

To leverage the strengths of the availability of both diagnosis of EDs and self‐reported ED symptoms among the DNBC‐18 participants, we defined a combined outcome measure of any EDs where individuals with either a diagnosed or self‐reported ED were classified as cases, and those without an ED served as the reference group. Additionally, we applied a hierarchical approach to distinguish between diagnosed and non‐diagnosed EDs, where a diagnosis overruled a self‐reported classification. This resulted in three mutually exclusive categories: (1) diagnosed EDs, (2) non‐diagnosed EDs, and (3) no EDs, with the latter serving as the reference category.

### 2.4 Statistical Analyses

We used descriptive statistics to assess the incidence of diagnosed EDs, the proportion of diagnosed and non‐diagnosed EDs in DNBC‐18, and the characteristics of the participants. We applied a group‐based multi‐trajectory model as in the DANLIFE study (Nagin et al. 2018; Rod et al. 2020) to identify distinct patterns of adversity across the three predefined dimensions: material deprivation, loss or threat of loss, and family dynamics during ages 0–9. Models with two to eight trajectory groups were fitted using the TRAJ package in Stata (version 14.2), applying zero‐inflated Poisson regressions with a cubic trajectory function of age to the annual adversity count within each dimension. This approach generated a probability for each individual to belong to a specific trajectory group. Individuals were assigned to the trajectory group with the highest probability of membership. The optimal number of trajectory groups was determined by evaluating model adequacy and ensuring that the groups were substantively distinct and of sufficient size. Given the multi‐trajectory context, we prioritized interpretability, as the selected model must be substantively meaningfull across all dimensions (Nagin et al. 2018). The DANLIFE study previously identified five trajectory groups based on data from individuals born between 1980 and 1998 and up to the age of 15 years. Since the age span and the birth years included in this study were different, we did not necessarily expect to identify the same number of trajectories.

For the first aim involving the total population, we applied a quasi‐Poisson regression to estimate the incidence rate ratio (IRR) and corresponding 95% confidence intervals (CI) for diagnosed EDs across the trajectory groups of childhood adversities, with the low adversity group as the reference. Time at risk began at age 10, with follow‐up ending at the earliest occurrence of death, emigration, diagnosed ED, or before reaching age 19. We conducted separate analyses for each ED subtype, including AN, BN, and EDNOS, allowing individuals to be classified under multiple subtypes. For overall ED diagnosis, analyses were stratified by sex, while subtype analyses were limited to females due to the insufficient number of male cases.

For the second aim involving DNBC‐18, we used logistic regressions to estimate the odds ratio (OR) and corresponding 95% CI for the combined measure of any EDs versus no EDs. For the composite outcome separating diagnosed ED, non‐diagnosed EDs, and no EDs, we applied multinomial logistic regressions to estimate the relative risk ratio (RRR) and corresponding 95% CI. We performed a likelihood ratio test to assess whether modeling the outcome with three categories, rather than as a binary measure, improved model fit. Due to the limited number of male cases, analyses were conducted for the full sample and subsequently for females alone.

All analyses were performed crude and adjusted, accounting for maternal and paternal age at birth, parental origin, urbanicity at birth, birth year, and parental education at birth (highest attained or ongoing). A detailed description of the covariates is presented in Table S2. Parental origin was only included in the adjustments for analyses involving the total population, as the number of non‐Danish individuals was too small to allow meaningful adjustment in the DNBC‐18. Covariates were selected based on prior evidence of their role as potential confounders of the association between childhood adversity and EDs (Larsen et al. 2017; Su et al. 2016). The analyses, including the DNBC‐18, were additionally weighted using inverse probability weighting (IPW) to address differential attrition (Nohr and Liew 2018). Participants with a lower probability of participation in DNBC‐18 were assigned larger weights, while those with a greater probability were assigned smaller weights, reflecting the characteristics of the background population (Seaman and White 2013). Participation probabilities were estimated using a logistic regression model with the following predictors: sex (assigned at birth), maternal and paternal age, urbanicity, and parity, all measured at birth. Highest parental education (attained or ongoing), equivalized household income, parental cohabitation status, out‐of‐home placement, childhood psychiatric diagnoses, and parental psychiatric diagnoses were assessed up to or at the child's age of 10 years. A detailed description of the predictors is presented in Table S3. To handle missing data in covariates used for IPW estimation, we included a separate missing category for each variable. This approach was chosen to retain participants with partial data and correct for differential participation, not to estimate causal effects. To avoid extreme weights, we stabilized them by multiplying by the overall probability of participating in the DNBC‐18, ensuring that the mean of the weights remained close to the overall participation rate. The standardized mean difference (SMD) was calculated to evaluate the balance of covariates between the weighted and unweighted samples. An SMD of less than 0.1 indicated an acceptable covariate balance (Stuart et al. 2013).

In a supplementary analysis, we examined the co‐occurrence of diagnosed EDs and self‐reported ED by calculating the percentage overlap for overall EDs and subtype‐specific EDs to assess potential heterogeneity in ED presentations. In a second supplementary analysis, we assessed potential selection bias by examining whether associations between trajectories of childhood adversities and diagnosed EDs differed across key population subsets: the total population (children born in Denmark, 1996–2003), the background population (live‐born children eligible for DNBC), those invited to DNBC‐18 (enrolled into DNBC and not opted out), and the unweighted and weighted DNBC‐18 samples. These subsets reflect stages in the selection process and were used to evaluate how participation may influence observed associations and whether the weighted DNBC‐18 estimates aligned with those in the background population. Additionally, we conducted five sensitivity analyses. First, we repeated all analyses without adjusting for parental education, following the approach used by DANLIFE, as parental education is highly correlated with material deprivation. Second, participants with a parental history of EDs up to the age of 10 were excluded to examine the potential impact of parental EDs, a component of the family dynamics dimension of childhood adversity, on the study findings. Third, for the first aim, including the total population, we stratified the birth years 1996–1999 versus 2000–2003 to investigate the potential temporal trends in the incidence rates of mental disorders (Momen et al. 2025; Plana‐Ripoll et al. 2022). Data were too sparse to explore this in the second aim. Fourth, for the second aim, we restricted the definition of non‐diagnosed EDs to individuals with self‐reported height and weight measured within 1 year from responding to the survey (*N* = 38,146), ensuring that any time gap in the BMI measurement did not influence the results. Finally, for the second aim, we restricted the non‐diagnosed EDs to include only self‐reported symptoms of AN and BN, allowing us to assess whether BED primarily drove the potential association. This sensitivity analysis also addressed the limitation that BED is not captured in register‐based diagnoses, thereby enabling a more consistent comparison between diagnosed and self‐reported ED outcomes.

## 3 Results

**FIGURE 1:**
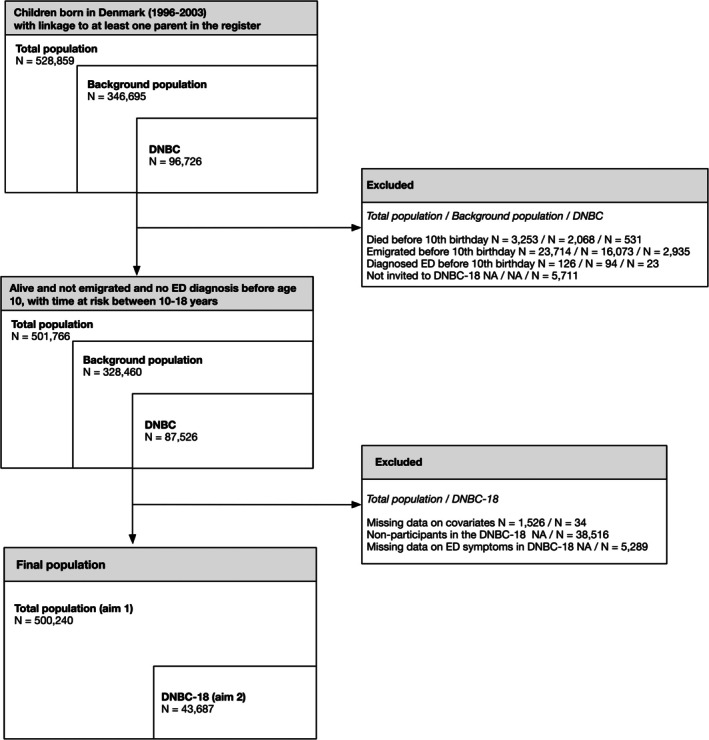
Study flow chart of the total population, background population in the Danish National Birth Cohort (DNBC), and the 18‐year follow‐up (DNBC‐18). Note that the sizes of the boxes are not proportional to the population size.

The first aim, encompassing the total population, resulted in a sample of 500,240 individuals (Figure 1). For the second aim, 43,687 participants (50% of the 87,526 eligible individuals, response rate: 56%) completed the DNBC‐18 survey and had information on all items related to self‐reported ED symptoms. This sample was weighted to represent the background population of 328,460 individuals (Figure 1).

### 3.1 Prevalence and Incidence of Eating Disorders

**FIGURE 2:**
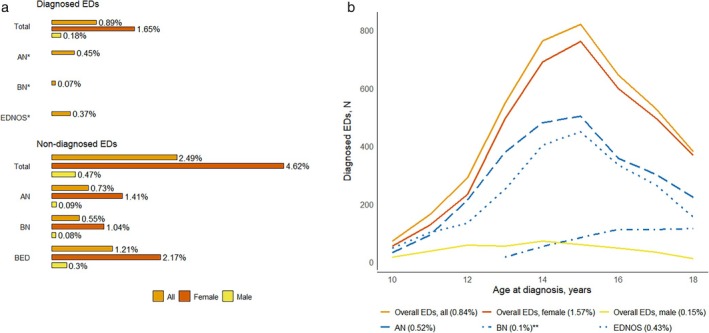
(a) Weighted proportion of diagnosed eating disorders (EDs) between 10 and 18 years and non‐diagnosed EDs in the DNBC‐18 (*N* = 43,687). (b) Incidence of diagnosed EDs between 10 and 18 years in the total population (*N* = 500,240), (%) shows the total incidence proportion having the specific ED between 10 and 18 years. *Too few observations to report for females and males. **Too few observations to report from ages 10 to 12 years.

Less than 1% of the population had a diagnosed ED (Figure 2a,b). In the DNBC‐18, all proportions were weighted. AN (0.45%, *N* = 243) was the most prevalent ED subtype (Figure 2a). The prevalence of non‐diagnosed EDs was 2.49% (*N* = 1201), with non‐diagnosed BED being the most common subtype (1.21%, *N* = 550). Sex differences were pronounced for both diagnosed EDs (1.65% in females, *N* = 428 vs. 0.18% in males, *N* = 31) and non‐diagnosed EDs (4.62% in females, *N* = 1119 vs. 0.47% in males, *N* = 82). Figure 2b depicts the incidence of diagnosed EDs between ages 10–18 in the total population, showing a peak at age 15. However, AN and EDNOS peak earlier, around age 14, while BN peaks later, around age 16.

### 3.2 Trajectories of Childhood Adversities

**FIGURE 3:**
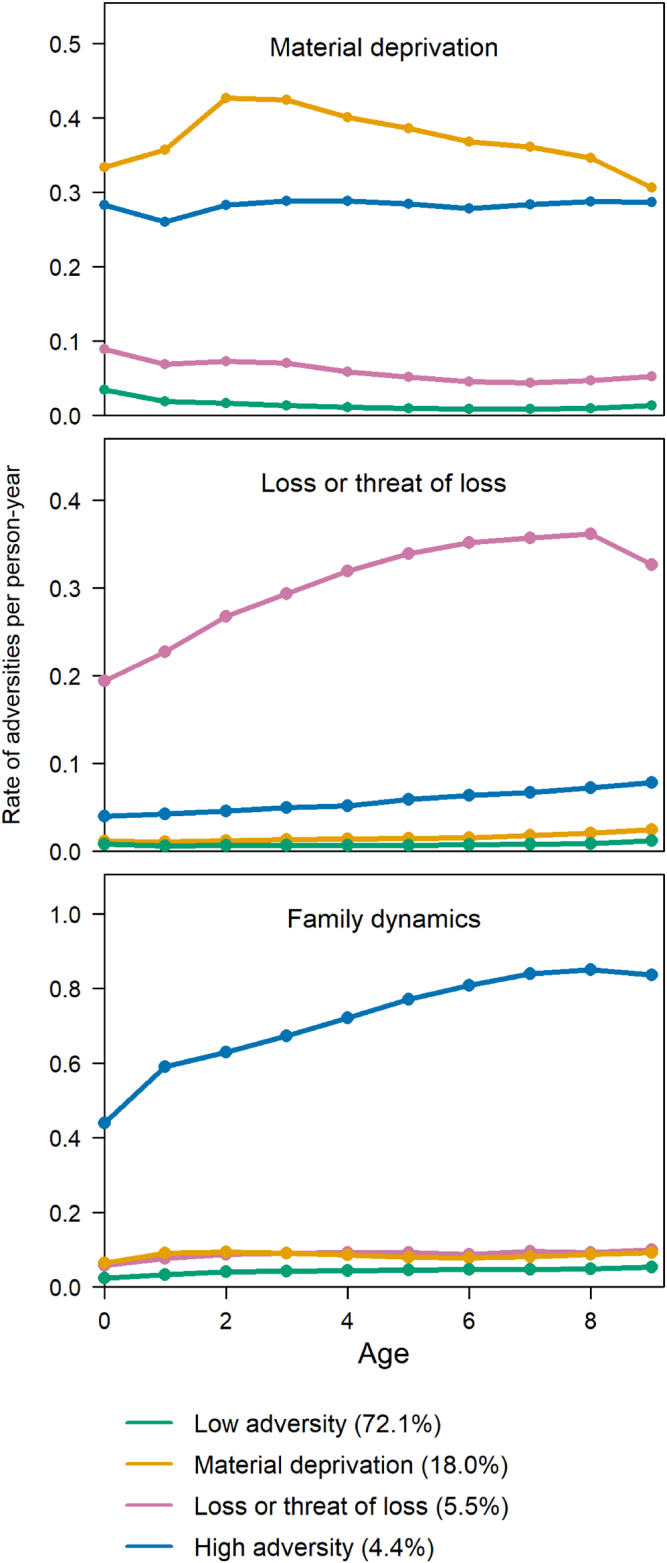
Estimated trajectory groups of childhood adversities in the total population (*N* = 500,240). The adversities were categorized into three predefined dimensions: Material deprivation (i.e., family poverty and parental long‐term unemployment), loss or threat of loss within the family (i.e., parental severe somatic illness, sibling severe somatic illness, and death of a parent or sibling), and family dynamics (i.e., parental separation, being placed in foster care, parental psychiatric illness, sibling psychiatric illness, and parental alcohol or drug abuse). The *y*‐axis scale varies across plots.

The trajectories were based on data from the total population (*N* = 500,240). Based on visual evaluation and substantive interpretability, the four‐group model of childhood adversity from ages 0–9 years was selected as optimal (Figure 3). This solution yielded distinct and meaningful groups of sufficient size and demonstrated good model fit (Table S4). The five‐group model did not add a substantively distinct group compared with the four‐group model.

The low adversity group, comprising 72% of the population (*N* = 361,179), was characterized by consistently low levels of adversity across all dimensions before age 10 (Figure 3). The material deprivation group, representing 18% of the population (*N* = 89,756), experienced persistently high levels of adversity in the material deprivation dimension, while adversity levels were low in the other two dimensions. The loss or threat of loss group, accounting for 6% of the population (*N* = 27,437), experienced relatively high adversity levels related to loss or threat of loss, with relatively low adversity in the other two dimensions. Finally, the high‐adversity group, constituting 4% of the population (*N* = 21,868), showed persistently elevated adversity levels across all dimensions, particularly in the family dynamics dimension, with slightly elevated levels in the loss or threat of loss dimension.

### 3.3 Characteristics Across Trajectories of Childhood Adversity

Table 2 outlines the characteristics of the total population and the DNBC‐18 across the four trajectory groups. The results from implementing IPW demonstrated that it effectively balanced the included predictors, with the SMD consistently below the 0.1 threshold, indicating minimal imbalance (Table S5). Notable differences were observed in parental age, with younger parental ages at birth (< 25 years) being more prevalent in the material deprivation and high‐adversity groups (Table 2). As anticipated, older parental ages at birth (≥ 35 years) were more frequent in the loss or threat of loss group. Parental origin varied across the groups, with 23% of non‐Danish parents in the material deprivation group and 10% or less in the other groups. A relatively higher proportion of individuals in the high adversity group and the material deprivation group resided in rural areas. Marked differences in parental educational level were also observed, with lower levels relatively more prevalent in the high adversity and material deprivation groups and higher parental education levels highest in the low adversity group.

**TABLE 2:** Characteristics at time of birth across the four estimated trajectory groups in the total population and DNBC‐18.

	Total population	DNBC‐18	Low (72.1%/76.1%)^a^		Material deprivation (18.0%/14.0%)^a^		Loss or threat of loss (5.5%/5.3%)^a^		High (4.4%/4.7%)^a^	
	%	%^b^	%	%^b^	%	%^b^	%	%^b^	%	%^b^
	*N* = 500,240	*N* = 43,687	*N* = 361,179	*N* = 35,857	*N* = 89,756	*N* = 4668	*N* = 27,437	*N* = 2169	*N* = 21,868	*N* = 993
Maternal age at birth										
< 25 years	15	14	12	11	24	22	14	13	33	38
25–29 years	36	36	38	37	34	33	33	33	30	27
30–34 years	34	35	36	36	28	29	34	36	24	22
≥ 35 years	15	16	15	16	14	16	19	18	13	13
Paternal age at birth										
< 25 years	7	6	5	5	11	11	7	6	17	18
25–29 years	27	26	27	26	26	25	23	23	26	26
30–34 years	36	37	38	38	31	31	33	35	27	26
≥ 35 years	29	30	29	30	30	31	36	36	28	29
Not registered	1	1	1	1	1	2	1	0	1	1
Parental origin										
Danish	92	^e^	96	^e^	77	^e^	90	^e^	92	^e^
Non‐Danish	8	^e^	4	^e^	23	^e^	10	^e^	8	^e^
Urbanicity at birth										
Capital	27	27	26	28	29	24	29	29	24	21
City municipality	13	12	12	12	15	13	12	11	12	10
Provincial city Municipality	23	23	24	24	20	21	23	23	24	25
Suburban municipality	16	15	17	15	14	15	16	17	15	15
Rural municipality	21	22	21	21	23	28	20	20	25	29
Birth year^c^										
1996–1999	51	36	50	36	54	36	48	34	47	33
2000–2003	49	64	50	64	46	64	52	66	53	67
Parental education at birth^d^										
Low	10	9	6	5	19	18	12	11	36	38
Medium	45	44	44	43	49	49	46	46	45	46
High	45	47	50	52	32	33	42	44	19	17

^a^ Proportion of total population/DNBC‐18 (weighted *N* = 43,414).

^b^ Weighted percentage for the DNBC‐18.

^c^ Birth year distribution differs, as DNBC recruitment began in one county in 1996 and expanded to nationwide in 1999.

^d^ Highest attained or ongoing parental education.

^e^ Too few observations to report.

### 3.4 Diagnosed Eating Disorders in the Total Population

**FIGURE 4:**
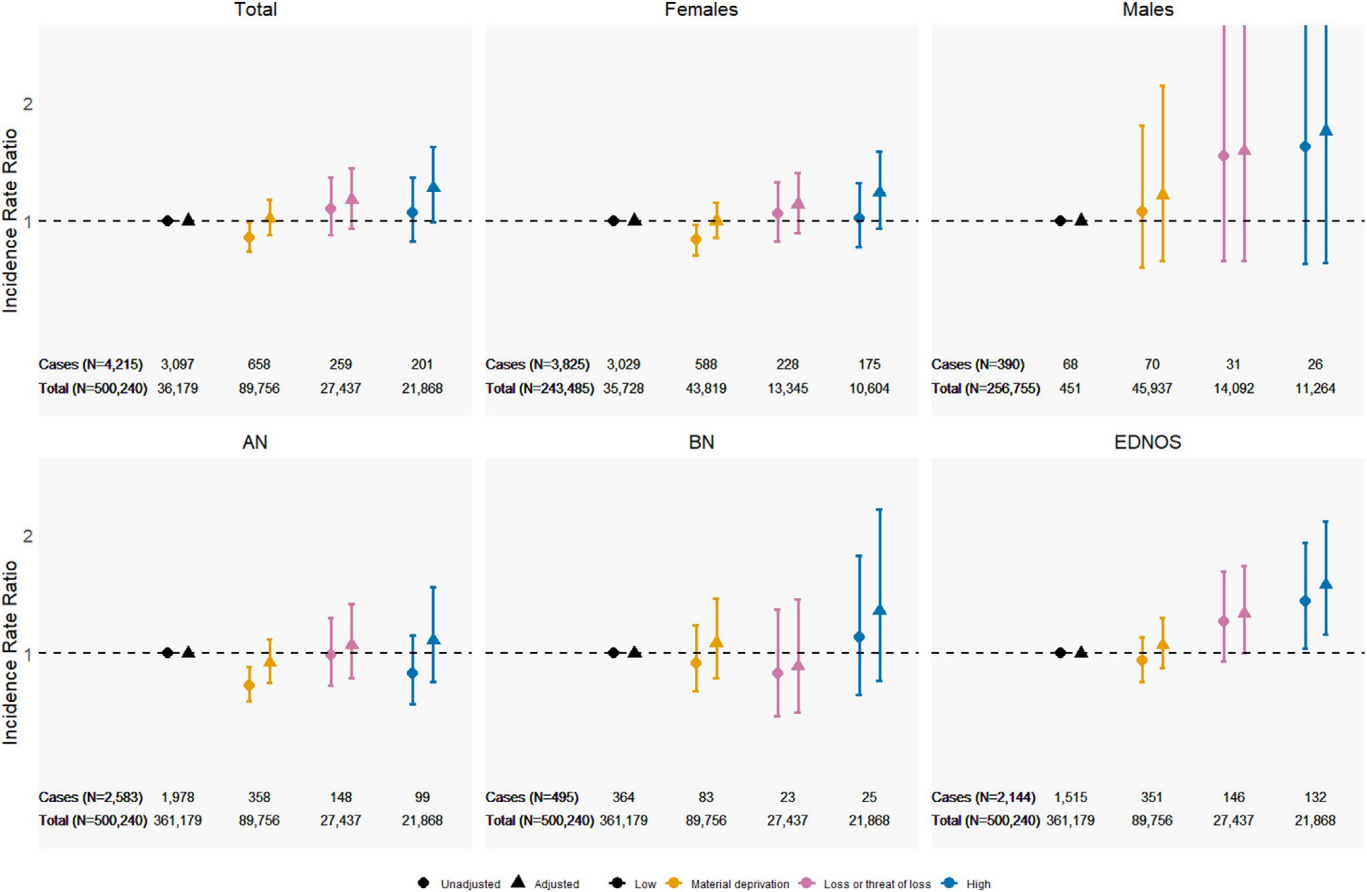
Trajectories of childhood adversities and diagnosed eating disorders (EDs) in the total population (*N* = 500,240). The figure presents the incidence rate ratio (IRR) with 95% confidence intervals (95% CI) for diagnosed EDs for each adversity group compared with the low adversity group. The top panel is for overall diagnosed EDs in the total population and stratified by sex (females and males). The bottom panel is for the subtypes: Anorexia nervosa (AN), bulimia nervosa (BN), and eating disorder not otherwise specified (EDNOS). Estimates are presented as crude and adjusted for maternal and paternal age at birth, parental origin, urbanicity at birth, birth year, and parental education (highest attained or ongoing) at birth. Results are also presented in Tables S6 and S7.

There were no differences in the IRRs for overall diagnosed EDs across the adversity groups, neither in the total population nor in the sex‐stratified analyses (Figure 4 and Table S6). The incidence of EDs was highest in the high adversity group (IRR = 1.28, 95% CI: 0.99–1.63; Figure 4 and Table S6), primarily driven by a higher incidence of EDNOS (Table S7). For EDNOS, the high adversity group showed a notably higher risk compared with the low adversity group (IRR = 1.58, 95% CI: 1.16–2.12; Table S7). No differences in AN or BN incidence were observed across adversity groups. However, in unadjusted models, the material deprivation group showed a lower risk of diagnosed AN compared to the low adversity group (Table S7), though this association attenuated after adjustment. When the analysis was restricted to females, the results remained largely consistent (Table S8).

### 3.5 Diagnosed and Non‐Diagnosed Eating Disorders in the DNBC‐18

**FIGURE 5:**
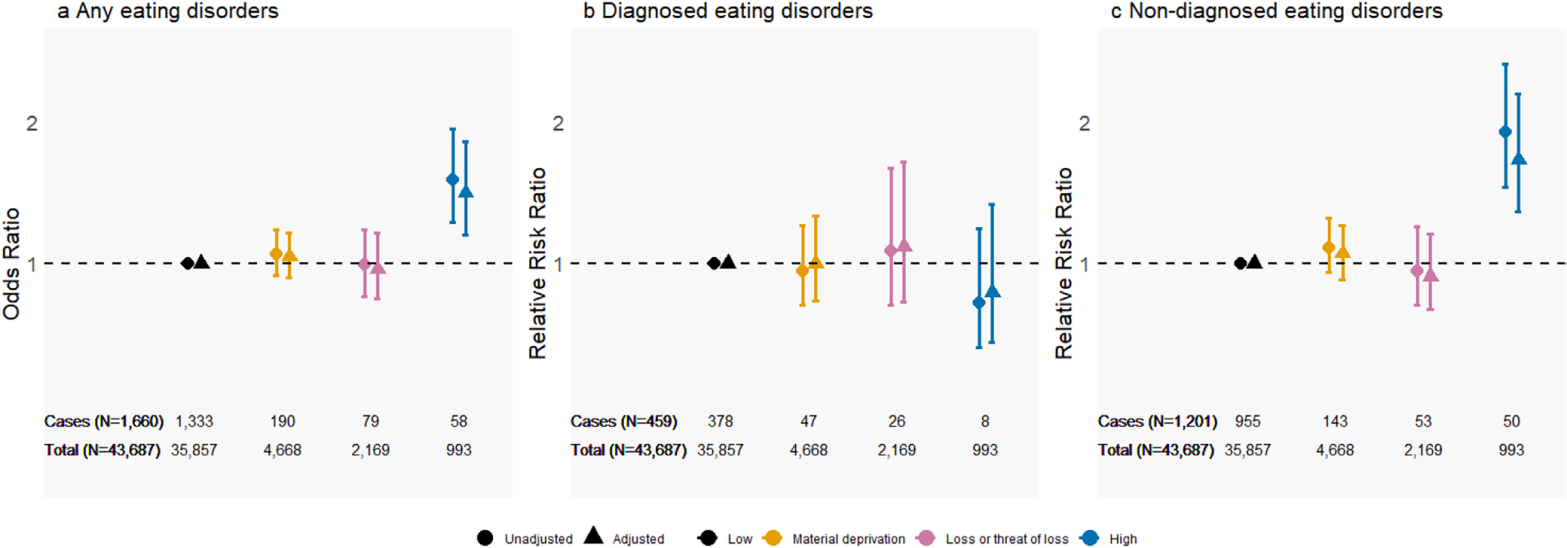
Association between trajectories of childhood adversities and eating disorders (EDs) in the DNBC‐18 (*N* = 43,687). (a) Odds ratio (OR) with 95% confidence for any EDs (combining diagnosed and self‐reported EDs as one group) versus no ED (reference group) compared with the low adversity group. 5b: Relative risk ratio (RRR) with 95% CI for a composite hierarchical outcome of diagnosed ED, non‐diagnosed EDs, and no ED (reference group) compared with the low adversity group. All estimates are presented as crude and adjusted for maternal and paternal age, urbanicity, birth year, and parental education (highest attained or ongoing) at birth. Results are also presented in Table S9. The likelihood ratio test showed that distinguishing diagnosed and non‐diagnosed EDs provided a better model fit than a binary outcome of any EDs (p = 0.001).

In the analysis of any EDs in DNBC‐18, the high adversity group had a higher risk of developing ED compared to the low adversity group (OR = 1.50, 95% CI: 1.20–1.86; Figure 5, Table S9). When distinguishing between diagnosed and non‐diagnosed EDs, no difference was observed in the risk of diagnosed EDs across adversity groups. However, for non‐diagnosed EDs, the high adversity group had a higher risk compared with the low adversity group (RRR = 1.73, 95% CI: 1.37–2.20). This increased risk was also seen among females only (RRR = 1.89, 95% CI: 1.48–2.43; Table S10).

### 3.6 Supplementary and Sensitivity Analyses

In the DNBC‐18, the co‐occurrence of diagnosed EDs and self‐reported EDs was 24% (Figure S1). Self‐reported BED symptoms showed the least overlap with diagnosed EDs (either AN, BN, or EDNOS), likely due to the absence of a distinct diagnostic category for BED in ICD‐10.

Applying IPW to balance DNBC‐18 with the background population yielded comparable estimates for low, material deprivation, loss or threat of loss groups, but for the high adversity group, the estimates were slightly different in the background population (IRR = 1.03, 95% CI: 0.78–1.33, Table S11) compared with the DNBC‐18 weighted population (IRR = 0.70, 95% CI: 0.26–1.51, Table S11). This difference may be attributed to random error, likely due to the small number of cases in the high adversity group.

In a sensitivity analysis omitting adjustments for parental education, the estimates for the total population were attenuated (Tables S6 and S7). In the DNBC‐18, the estimates for any EDs and non‐diagnosed EDs in the high adversity group showed slight amplification (Table S9), while the findings remained aligned with the main results. In another sensitivity analysis, excluding participants with a parental ED history before age 10, the estimates for diagnosed EDs in the total population were slightly higher (Table S12). In the DNBC‐18, the estimates for non‐diagnosed EDs in the high adversity group were attenuated (Table S13). Stratifying the analysis by birth year in the total population indicated a slightly higher risk for individuals born between 2000 and 2003 compared with 1996–1999 (Table S14), while the overall findings remained consistent. When restricting the DNBC‐18 analysis to cases where BMI in self‐reported ED definitions was based on weight and height measured within the past year, the estimates were attenuated (Table S15). Lastly, excluding BED from the non‐diagnosed EDs in the DNBC‐18 attenuated the results but did not fully account for the observed differences (Table S16).

## 4 Discussion

This study demonstrates that exposure to high levels of childhood adversity is associated with a higher risk of ED in adolescence, particularly non‐diagnosed EDs. Using comprehensive national register and cohort data, we identified four distinct trajectories of adversity from ages 0–9. While most children experienced low adversity, a smaller group was exposed to persistent and multidimensional adversity across multiple domains. These early‐life patterns provide important context for understanding disparities in ED diagnosis and access to care. In the total population, including all children born between 1996 and 2003, individuals in the high adversity group had a higher risk of being diagnosed with EDNOS compared to those in the low adversity group, while no associations were observed for other diagnosed EDs. Using DNBC‐18, we found that children in the high adversity group had a higher risk of any EDs, entirely driven by non‐diagnosed cases. These findings remained consistent when analyses were restricted to females. Sensitivity analyses, excluding non‐diagnosed BED in the DNBC‐18, attenuated the estimates but still showed a higher risk of non‐diagnosed EDs, including only AN and BN, among children in the high adversity group. These findings highlight the distinction between diagnosed and non‐diagnosed EDs, underscoring inequalities in diagnosis and access to treatment.

Our findings based on the DNBC‐18 align with previous population‐based studies that have reported associations between self‐reported childhood adversities and ED symptoms, including symptoms of AN, BN, and BED (Bodell et al. 2011; Copeland et al. 2015; Johnson et al. 2002; Loth et al. 2008; Thomas et al. 2020). However, the definitions of ED symptoms vary across studies. In our first aim, including the total population, our findings for EDNOS were consistent with those of a Danish register‐based study, which reported that childhood adversities were associated with an increased risk of EDNOS (Larsen et al. 2017). However, this previous study also found a higher risk of BN and a lower risk of AN, which we did not observe. The discrepancies may be attributed to differences in follow‐up periods. Specifically, our shorter follow‐up period likely limited our ability to capture the full spectrum of ED cases, particularly BN, which has a later onset. Another important difference is the measurement of childhood adversities. By employing a trajectory‐based approach, our study extends previous research by considering the timing, duration, and cumulative nature of adversities during early childhood. Previous studies have only focused on single adversities or did not account for the multi‐dimensional nature of adversity exposure. Our findings highlight the importance of considering cumulative adversities, as prolonged exposure to multiple stressors may have a more profound impact on the development of EDs.

Aligned with other studies, females consistently showed higher rates of both diagnosed and non‐diagnosed EDs, highlighting their higher risk for EDs compared with males. However, this disparity may partly result from diagnostic criteria traditionally aligned with female presentations of EDs, as well as differences in treatment‐seeking behavior and societal underrecognition of symptoms more common in males (Zayas et al. 2018). While clinical samples of diagnosed ED report a female‐to‐male ratio as high as 9:1, population‐based estimates suggest a lower ratio of approximately 2–3:1 (Swanson and Field 2016). Contrary to expectations, diagnosed and self‐reported cases showed a similar ratio, likely due to our operational definitions aligning with ED diagnostic thresholds. Using stringent criteria for self‐reported EDs improved comparability with diagnosed cases, making them more reflective of clinical presentations. Unlike other register‐based studies, we included males, but the low ED prevalence and limited adversity exposure in this group precluded sex‐stratified analyses.

A key strength of this study is the linkage of register‐based and self‐reported EDs in a large, population‐based sample followed from birth to adulthood. Embedding DNBC within the full Danish Birth cohort enabled identical register‐based measures of adversity and diagnosed EDs across samples. A limitation is self‐selection and attrition bias, as DNBC‐18 is a selective subset. Associations between childhood adversity and diagnosed EDs varied slightly by population: the estimate for high adversity was elevated in the invited population but attenuated in DNBC‐18 (Table S11), likely due to selective enrollment and dropout. We applied sample weights and tested alternative weighting via random forest models, though this did not improve alignment. Weighted estimates remained imprecise, likely due to the low number in the high‐adversity group and few ED cases in DNBC‐18, resulting in substantial statistical uncertainty across both weighted and unweighted models.

Using registries to measure childhood adversities is a strength, but they captured a limited range of adversities. Child abuse, neglect, and domestic violence were not directly measured, though severe cases may have been partially reflected in indicators like foster care placement. Family dynamics, such as parental separation and parental psychiatric illness, provided insight into the home environment but did not capture unmeasured factors like high‐conflict settings. Parental alcohol abuse was also likely underreported, relying on hospitalization and medication records. While the use of multiple, repeated indicators allowed us to identify general patterns, the full impact of childhood adversities may still be underestimated. A key strength of using self‐reported measures of self‐reported EDs is our ability to capture both clinical cases and individuals who may not engage with formal treatment, offering a broader perspective on the ED spectrum. However, this approach has limitations, including potential reporting bias and the fact that self‐reported and diagnosed EDs do not fully overlap in time. Self‐reports reflect the past year, whereas diagnosed EDs include any diagnosis recorded from age 10–18. Children referred to outpatient child and adolescent psychiatric care in Denmark have been shown to have struggled for years with their problems prior to referral, particularly in the case of neurodevelopmental disorders compared to emotional disorders, including EDs (Hansen et al. 2021). The age of the first‐time diagnosis was not our primary interest; instead, we applied a hierarchical approach to distinguish between diagnosed and non‐diagnosed cases, in which a diagnosis overruled self‐reported symptoms fulfilling diagnostic criteria for an ED. Thus, individuals who experienced ED symptoms between the ages of 10–18 but no longer report these at age 18 appear to have recovered without formal secondary care treatment. Moreover, while BED was included in the self‐reported outcome, it is not captured in register‐based diagnoses in ICD‐10, where such cases are typically classified as EDNOS (Larsen et al. 2017). Although the self‐reported ED items have not been formally validated in a Danish context, they are based on internationally recognized instruments and have previously been used in Danish adolescent cohorts, supporting their relevance for this study (Micali et al. 2015; Olsen et al. 2021). Additionally, as the register‐based ED diagnoses reflect hospital‐treated cases, those diagnosed only in private practice were not captured, potentially underestimating the true prevalence of diagnosed EDs, particularly among the less severe cases. A sensitivity analysis excluding BED from the self‐reported outcome yielded attenuated, yet consistent, associations for non‐diagnosed EDs, supporting the robustness of the findings.

We measured adversities from ages 0–9 to capture trajectories up to middle childhood and to ensure temporal separation from the typical onset of EDs. Although diagnosed EDs are rare before age 10, non‐diagnosed cases may occur but cannot be reliably identified in the available data (Larsen et al. 2024; Momen et al. 2025; Nicholls et al. 2011). While this design aims to ensure temporal ordering between exposure and outcome, we cannot fully rule out reverse causation, as stress related to a child suffering from an ED or a severe feeding disorder could plausibly influence family dynamics or economic stability.

This is the first study to link individual‐level data on diagnosed and non‐diagnosed EDs. Integrating self‐reported data with hospital records provides a more comprehensive understanding of ED prevalence and characteristics from both clinical and self‐reported perspectives. These findings highlight that childhood adversities are associated with a higher risk of diagnosed EDNOS and a higher risk of non‐diagnosed EDs, underscoring the need for targeted support for high‐risk groups. Importantly, the results highlight significant diagnostic gaps that may stem from differences in symptom recognition, treatment‐seeking behavior, and access to care. These gaps persist even in a tax‐funded health system offering free hospital‐based treatment, underscoring the need for more targeted efforts. Individuals in the high adversity group were at higher risk of being undiagnosed, indicating a critical gap in early identification and access to appropriate intervention. One promising approach is the integration of structured adversity screening into primary care or school health settings. As supported by previous research highlighting the potential of early adversity screening to identify unmet psychosocial needs and improve access to supportive services (Gottlieb et al. 2016), such efforts could help detect at‐risk children earlier and enable more timely and equitable support.

## 5 Conclusion

This study demonstrates that cumulative childhood adversities are associated with a higher risk of EDs, particularly among those not captured by clinical diagnoses. By linking self‐reported and registry data, we uncover persistent diagnostic gaps, likely reflecting differences in symptom recognition, help‐seeking, and access to care. These findings highlight the need for early detection efforts and targeted prevention in high‐risk groups.

## Author Contributions

**Andrea Joensen:** conceptualization, investigation, writing – original draft, methodology, data curation, formal analysis. **Leonie K. Elsenburg:** methodology, supervision, writing – review and editing, investigation, data curation. **Else Marie Olsen:** writing – review and editing, supervision, investigation. **Claus Thorn Ekstrøm:** methodology, investigation, conceptualization, writing – review and editing, visualization, supervision. **Naja Hulvej Rod:** investigation, writing – review and editing, methodology. **Katrine Strandberg‐Larsen:** conceptualization, funding acquisition, investigation, methodology, writing – review and editing, visualization, supervision.

## Ethics Statement

The DNBC cohort is approved by the Danish Data Protection Agency under the general approval granted to Statens Serum Institut (fællesfortegnelse), reference number 18/04608 and by the Committee on Health Research Ethics (case number (KF) 01‐471/94). Participants in the DNBC were enrolled with informed consent and individuals born into the cohort were notified about their participation, rights, and the option to opt out upon turning 18. Data approval for the analyses conducted in this study, which involved linking DNBC data with register data accessed on Statistics Denmark's server, was obtained from the DNBC managerial team (2018‐15), Statistics Denmark and registered under the Danish Data Protection Agency's general approval for the Faculty of Health and Medical Sciences at the University of Copenhagen (514‐0400/19‐3000).

## Conflicts of Interest

The authors declare no conflicts of interest.

## Supporting information


**Data S1:** Supporting Information.

## Data Availability

The data that support the findings of this study are available from the corresponding author upon reasonable request.
