# Supplementary material for: Trajectories of Childhood Adversity and Eating Disorders in Adolescence
Source: Int J Eat Disord. 2025 Jul 28;58(11):2077–89. doi: 10.1002/eat.24514 (PMC12605683; doi:10.1002/eat.24514)
Supplement: Supplementary file 1 — Data S1: Supporting Information. [file EAT-58-2077-s001.docx]

# Supplementary

# **Trajectories of childhood adversity and eating disorders in adolescence**

Andrea Joensen ([0000-0003-1877-9174](https://orcid.org/0000-0003-1877-9174)), Leonie K. Elsenburg ([0000-0002-9824-9837](https://orcid.org/0000-0002-9824-9837)), Else Marie Olsen ([0000-0002-7240-9767](https://orcid.org/0000-0002-7240-9767)), Claus Thorn Ekstrøm ([0000-0003-1191-373X](https://orcid.org/0000-0003-1191-373X)), Naja Hulvej Rod ([0000-0002-6400-5105](https://orcid.org/0000-0002-6400-5105)), Katrine Strandberg-Larsen ([0000-0001-7061-3767](https://orcid.org/0000-0001-7061-3767))

Supplementary

[Table S1: Definitions of childhood adversities by DANLIFE with adaptions 1](#_Toc192789686)

[Table S2: Definitions of covariates, measured at birth 3](#_Toc192789687)

[Table S3: Definitions of predictors for inverse probability weighting (IPW) in the 18-year follow-up in the Danish National Birth Cohort 18-year follow-up (DNBC-18) 4](#_Toc192789688)

[Table S4: Model Adequacy and Fit Statistics for the Selection of 2- to 8-Group Trajectory Models Using Average Posterior Probability, Bayesian Information Criterion (BIC), and Group Membership Probability 5](#_Toc192789689)

[Table S5: Predictors ^a^ included for inverse probability weighting (IPW) across the total, background, invited to 18-year follow-up in the Danish National Birth Cohort (DNBC-18), unweighted and weighted and weighted DNBC-18 population. 6](#_Toc192789690)

[Table S6: Incidence Rate Ratio (IRR) and 95% confidence interval (95% CI) for diagnosed eating disorders (EDs), overall and stratified by sex, across trajectories of childhood adversities in the total population (N=500,240) 7](#_Toc192789691)

[Table S7: Incidence Rate Ratio (IRR) and 95% confidence interval (95% CI) for diagnosed eating disorder subtypes anorexia nervosa (AN), bulimia nervosa (BN) and eating disorders not otherwise specified (EDNOS), across trajectories of childhood adversities in the total population (N=500,240) 8](#_Toc192789692)

[Table S8: Incidence Rate Ratio (IRR) and 95% confidence interval (95% CI) for diagnosed eating disorder subtypes anorexia nervosa (AN), bulimia nervosa (BN) and eating disorders not otherwise specified (EDNOS), across trajectories of childhood adversities restricted to females only in the total population (N=243,485) 9](#_Toc192789693)

[Table S9: Odds ratio (OR) and 95% confidence interval (95% CI) for any eating disorders (EDs), including both diagnosed and threshold EDs, and relative risk ratio (RRR) and 95% CI for diagnosed EDs and non-diagnosed EDs across trajectories of childhood adversities in the 18-year follow-up in the Danish National Birth Cohort (DNBC-18; N=43,687) 10](#_Toc192789694)

[Table S10: Relative Risk Ratio (RRR) and 95% confidence interval (95% CI) for diagnosed eating disorders (ED) and non-diagnosed EDs across trajectories of childhood adversities, restricted to females only in the 18-year follow-up in the Danish National Birth Cohort (DNBC-18; N=25,636) 11](#_Toc192789695)

[Table S11: Incidence Rate Ratio (IRR) and 95% confidence interval (95% CI) for diagnosed eating disorders (ED) and trajectories of childhood adversities across the total population, background population, invited to the 18-year follow-up in the Danish National Birth Cohort (DNBC-18) and DNBC-18 weighted and unweighted. Estimates are presented as unadjusted. 12](#_Toc192789696)

[Table S12: Incidence Rate Ratio (IRR) and 95% confidence interval (95% CI) for diagnosed eating disorders (EDs) across trajectories of childhood adversities in the total population, restricted to no parental EDs before the child’s 10^th^ birthday (N=497,342) 13](#_Toc192789697)

[Table S13: Relative Risk Ratio (RRR) and 95% confidence interval (95% CI) for diagnosed eating disorders (EDs) and non-diagnosed EDs across trajectories of childhood adversities in the total population, restricted to no parental EDs before the child’s 10^th^ birthday (N=43,453) 14](#_Toc192789698)

[Table S14: Incidence Risk Ratio (IRR) and 95% confidence interval (95% CI) for diagnosed eating disorders (EDs) across trajectories of childhood adversities stratified by birth year 1996 to 1999 and 2000 to 2003 in the total population (N=500,240) 15](#_Toc192789699)

[Table S15: Relative Risk Ratio (RRR) and 95% confidence interval (95% CI) for diagnosed eating disorders (EDs) and non-diagnosed EDs across trajectories of childhood adversities, restricted to information on weight and height (body mass index measurement) measured within the past year in the 18-year follow-up in the Danish National Birth Cohort (DNBC-18; N=38,146) 16](#_Toc192789700)

[Table S16: Relative Risk Ratio (RRR) and 95% confidence interval (95% CI) for diagnosed eating disorders (EDs) and non-diagnosed EDs across trajectories of childhood adversities, restricted to only threshold anorexia nervosa (AN) and bulimia nervosa (BN) cases in the 18-year follow-up in the Danish National Birth Cohort (DNBC-18; N=43,137) 17](#_Toc192789701)

[Figure S1: Figure depicting co-occurrence of diagnosed eating disorders (EDs) and threshold EDs, including subtypes of threshold anorexia nervosa (AN), bulimia nervosa (BN), and binge eating disorder (BED) and diagnosed AN, BN, and eating disorder not otherwise specified (EDNOS) in the 18-year follow-up in the Danish National Birth Cohort (DNBC-18; N=43,687) 18](#_Toc192789702)

| **Table S1**: Definitions of childhood adversities by DANLIFE with adaptions | | | | | |
| --- | --- | --- | --- | --- | --- |
|  | | **Definitions** | **Registers** | | **Adaptions** |
| **Material deprivation** | | | | | |
| Family poverty | One count per year of life when the family income is lower than 50% of the median national family income | | The Income Register | aekvivadisp_13 | No adaptions |
| Long-term unemployment | One count per year of life for each parent being unemployed for at least 12 months | | The Integrated Database for Labor Marked Affiliation | Arledgr (1996-2008)  Arledgr (from 2008-2018) | No adaptions |
| **Loss or threat of loss** | | | | | |
| Death of a parent | One count in the year a parent dies | | Danish Civil Registration system | Date of death | No adaptions |
| Death of a sibling | One count for each death of a sibling | | Danish Civil Registration system | Date of death | Half siblings are also included |
| Parent somatic illness | One count per year of life for each parent diagnosed with one of the illnesses related to mortality included in the Charlson comorbidity index | | The Danish National Patient Register | All diagnosis codes listed in Thygesen et al 2011^35^ | Action and secondary diagnosis are included, and all hospital contacts are counted as an event. In DANLIFE, all hospital contacts are also included from 1995 and onwards (ICD-10) |
| Sibling somatic illness | One count per year of life for each sibling diagnosed with one of the seven most common somatic illnesses related to mortality in children aged 0–18 years in Denmark: malignant neoplasm, congenital anomalies of the heart and circulatory system, congenital anomalies of the nervous system, cerebral palsy, epilepsy, cardiomyopathy, and congenital disorders of lipid metabolism | | The Danish National Patient Register | C00-C96, Q20-Q28, Q00-Q07, G80-G83, G40-G41, I42-I43, E75, | Action and secondary diagnosis are included, and all hospital contacts are counted as an event. Half siblings are also included |

| **Family dynamics** | | | | |
| --- | --- | --- | --- | --- |
| Foster care | One count per year of life overlapping with a calendar year in which the child was registered as placed in out-of-home care | The Register of Support for Children and Adolescents | Effektdato  Haendelse | No adaptions |
| Parental psychiatric illness | hospital admission with a diagnosis related to psychiatric illness (excluding main diagnoses related to alcohol and drug abuse) | The Danish Psychiatric Central Research Register | All F-diagnosis are included expect diagnosis related to alcohol or drug abuse (same diagnosis codes used to define the variables parental alcohol abuse and parental drug abuse). Diagnosis code for tobacco abuse is also excluded. | Action and secondary diagnosis are included, and all hospital contacts are counted as an event |
| Sibling psychiatric illness | One count per year of life for each sibling with a hospital admission with a diagnosis related to psychiatric illness | The Danish Psychiatric Central Research Register | All F-diagnosis are included. No diagnoses are excluded. | Action and secondary diagnosis are included, and all hospital contacts are counted as an event. Half siblings are also included |
| Parental alcohol abuse | One count per year of life for each parent diagnosed with an illness related to alcohol abuse or receiving a prescription of a drug used in treatment of alcohol addiction | The Danish Psychiatric Central Research Register, the Danish National Patient Register, and the Danish National Prescription Registry | F10, F70, G621, I1426, K852, K860, K292  N07BB (N07BB01 N07BB2 N07BB3 N07BB4 N07BB5) | No adaptions |
| Parental drug abuse | One count per year of life for each parent diagnosed with an illness related to drug abuse or receiving a prescription of drugs used in treatment of drug addiction | The Danish Psychiatric Central Research Register, the Danish National Patient Register, and the Danish National Prescription Registry | F11-F16, F18, F19  N07BC (N07BC01, N07BC02, N07BC03, N07BC04, N07BC05, N07BC06, N07BC51) | No adaptions |
| Parental separation | One count per year of life overlapping with a calendar year in which the parents were separated | The Danish Civil Registration System | E_faelle_id | No adaptions |

| **Table S2:** Definitions of covariates, measured at birth | | |
| --- | --- | --- |
| **Covariate** | **Source** | **Definitions** |
| Maternal age at birth | Danish Medical Birth Register | <25, 25-29, 30-34, ≥35 |
| Paternal age at birth | Danish Medical Birth Register | <25, 25-29, 30-34, ≥35,  not registered ^a^ |
| Parental origin | Population Register | Danish, non-Danish |
| Urbanicity at birth | Population Register | Capital, metropolitan municipality, provincial municipality, suburban municipality, rural municipality |
| Birth year | Danish Medical Birth Register | 1996-1999, 2000-2003 |
| Parental education ^b^ | Danish Education Registers | Low, medium, high |
| ^a^ If paternal age at birth was missing, we classified these cases as 'not registered', as it is likely that no father was registered rather than the age simply being missing. This approach prevents the exclusion of these cases from the analysis  ^b^ Parental education (highest attained or ongoing, measured at birth. Low parental education covers short (primary and lower secondary), medium (upper secondary and post-secondary non-tertiary), and high (tertiary education) | | |

| **Table S3:** Definitions of predictors for inverse probability weighting (IPW) in the 18-year follow-up in the Danish National Birth Cohort 18-year follow-up (DNBC-18) | | |
| --- | --- | --- |
| Predictors | Source | Definitions |
| Sex (assigned at birth) ^a^ | Danish Medical Birth Register | Male, female |
| Maternal age at birth (years) ^a^ | Danish Medical Birth Register | <25, 25-29, 30-34, ≥35 |
| Paternal age at birth (years) ^a, b^ | Danish Medical Birth Register | <25, 25-29, 30-34, ≥35, not registered |
| Urbanicity at birth ^a^ | Population Register | Capital, metropolitan municipality, provincial municipality, suburban municipality, rural municipality |
| Parity ^a^ | Danish Medical Birth Register | 1, 2, ≥3 |
| Parental education ^c^ | Danish Education Registers | High, medium, low |
| Equivalized household income ^d, e^ | Income Register | Q1, Q2, Q3, Q4 |
| Parental cohabitation ^d^ | Danish Civil Registers | Yes, no |
| Out-of-home placement ^d^ | The Register of Support for Children and adolescents | Yes, no |
| Psychiatric diagnosis in childhood^33^ ^f^ | The Danish National Patient Register | Yes, no |
| Parental psychiatric diagnosis^33^ ^f^ | The Danish National Patient Register | Yes, no |
| ^a^ Measured at birth  ^b^ If paternal age at birth was missing, we classified these cases as 'not registered', as it is likely that no father was registered rather than the age simply being missing. This approach prevents the exclusion of these cases from the analysis  ^c^ Parental education (highest attained or ongoing), measured at age 10 years. Low parental education covers short (primary and lower secondary), medium (upper secondary and post-secondary non-tertiary), and high (tertiary education)  ^d^ Measured at the year the child turned 10 years  ^e^ Equivalized household income is adjusted for family size and divided into year-specific quartiles to account for inflation and the eight-year birth period of DNBC participants  ^f^ Measured up until the child turned 10 years | | |

| **Table S4:** Model Adequacy and Fit Statistics for the Selection of 2- to 8-Group Trajectory Models Using Average Posterior Probability, Bayesian Information Criterion (BIC), and Group Membership Probability. | | | | | | | | |
| --- | --- | --- | --- | --- | --- | --- | --- | --- |
| **Number of groups** | | **2** | **3** | **4** | **5** | **6** | **7** | **8** |
| **Average posterior probability** | | |  |  |  |  |  |  |
|  | 1 | 0.96 | 0.89 | 0.94 | 0.93 | 0.92 | 0.91 | 0.83 |
|  | 2 | 0.91 | 0.95 | 0.89 | 0.88 | 0.92 | 0.84 | 0.81 |
|  | 3 |  | 0.87 | 0.85 | 0.79 | 0.70 | 0.79 | 0.83 |
|  | 4 |  |  | 0.89 | 0.85 | 0.78 | 0.67 | 0.62 |
|  | 5 |  |  |  | 0.92 | 0.85 | 0.87 | 0.87 |
|  | 6 |  |  |  |  | 0.92 | 0.79 | 0.80 |
|  | 7 |  |  |  |  |  | 0.92 | 0.79 |
|  | 8 |  |  |  |  |  |  | 0.93 |
|  |  |  |  |  |  |  |  |  |
| **BIC – based on no data points ^a,c^** | | -337567.45 | -330576.66 | -325473.66 | -322688.90 | -321413.35 | -321481.56 | -320880.50 |
| **BIC- based on sample size ^b,c^** | | -337504.52 | -330481.43 | -325346.11 | -322529.05 | -321221.18 | -321257.08 | -320623.71 |
| **Group of membership probability (SE)** | | | |  |  |  |  |  |
|  | 1 | 74.85 (0.27) | 72.65 (0.27) | 69.62 (0.29) | 65.93 (0.33) | 62.34 (0.39) | 63.98 (0.36) | 49.46 (0.51) |
|  | 2 | 25.15 (0.27) | 18.71 (0.27) | 19.29 (0.27) | 17.40 (0.26) | 12.37 (0.23) | 13.49 (0.27) | 23.88 (0.37) |
|  | 3 |  | 8.64 (0.20) | 6.30 (0.18) | 9.45 (0.29) | 10.55 (0.35) | 13.49 (0.27) | 8.25 (0.27) |
|  | 4 |  |  | 4.79 (0.14) | 5.72 (0.16) | 7.79 (0.24) | 5.14 (0.17) | 6.70 (0.40) |
|  | 5 |  |  |  | 1.49 (0.07) | 5.63 (0.16) | 5.10 (0.24) | 5.07 (0.17) |
|  | 6 |  |  |  |  | 1.29 (0.06) | 1.75 (0.11) | 4.07 (0.17) |
|  | 7 |  |  |  |  |  | 1.32 (0.06) | 1.72 (0.11) |
|  | 8 |  |  |  |  |  |  | 0.82 (0.05) |
| ^a^ Calculated without directly incorporating the sample size. It assesses model fit based on likelihood and complexity without adjusting for the number of observations  ^b^ Incorporates the sample size in its calculation, applying a stronger penalty for complex models. This helps in selecting the best-fitting model while balancing fit and simplicity  ^c^ BIC scores closer to zero indicate better model fit | | | | | | | | |

|  |
| --- |

| **Table S5:** Predictors ^a^ included for inverse probability weighting (IPW) across the total, background, invited to 18-year follow-up in the Danish National Birth Cohort (DNBC-18), unweighted and weighted DNBC-18 population. | | | | | | | | | | | | |
| --- | --- | --- | --- | --- | --- | --- | --- | --- | --- | --- | --- | --- |
|  | **Total population** |  | **Background  Population** |  | **Invited to  DNBC-18** |  | **DNBC-18**  **unweighted** |  | **DNBC-18 Weighted** |  | **Standardized mean difference (SMD)** |  |
|  | **N = 501,766** |  | **N = 328,460** |  | **N = 87,526** |  | **N = 43,687** |  | **N = 43,687** |  |  |  |
|  | **%** |  | **%** |  | **%** |  | **%** |  | **%** |  |  |  |
| **Sex (assigned at birth)** |  |  |  |  |  |  |  |  |  |  |  |  |
| Male | 51 |  | 51 |  | 51 |  | 41 |  | 51 |  | 0.002 |  |
| Female | 49 |  | 49 |  | 49 |  | 59 |  | 49 |  |  |  |
| **Maternal age (years)** | |  |  |  |  |  |  |  |  |  |  |  |
| <25 | 15 |  | 14 |  | 10 |  | 8 |  | 14 |  | 0.002 |  |
| 25-29 | 36 |  | 36 |  | 38 |  | 38 |  | 36 |  |  |  |
| 30-34 | 34 |  | 34 |  | 37 |  | 38 |  | 35 |  |  |  |
| ≥35 | 15 |  | 15 |  | 15 |  | 15 |  | 16 |  |  |  |
| missing | <0.5 |  | <0.5 |  | - |  | - |  | - |  |  |  |
| **Paternal age(years)** | |  |  |  |  |  |  |  |  |  |  |  |
| <25 | 7 |  | 7 |  | 4 |  | 4 |  | 7 |  | 0.002 |  |
| 25-29 | 27 |  | 26 |  | 27 |  | 26 |  | 26 |  |  |  |
| 30-34 | 36 |  | 36 |  | 39 |  | 39 |  | 37 |  |  |  |
| ≥35 | 29 |  | 30 |  | 29 |  | 30 |  | 30 |  |  |  |
| not registered | 1 |  | 1 |  | <0.5 |  | <0.5 |  | 1 |  |  |  |
| **Parity** |  |  |  |  |  |  |  |  |  |  |  |  |
| 1 | 42 |  | 42 |  | 45 |  | 47 |  | 42 |  | 0.002 |  |
| 2 | 36 |  | 36 |  | 36 |  | 35 |  | 37 |  |  |  |
| ≥3 | 19 |  | 19 |  | 16 |  | 15 |  | 18 |  |  |  |
| missing | 2 |  | 3 |  | 3 |  | 4 |  | 3 |  |  |  |
| **Urbanicity** |  |  |  |  |  |  |  |  |  |  |  |  |
| Capital | 27 |  | 29 |  | 23 |  | 23 |  | 27 |  | 0.011 |  |
| City municipality | 13 |  | 12 |  | 14 |  | 14 |  | 12 |  |  |  |
| Provincial city municipality | 23 |  | 23 |  | 26 |  | 26 |  | 23 |  |  |  |
| Suburban municipality | 16 |  | 15 |  | 17 |  | 17 |  | 15 |  |  |  |
| Rural municipality | 21 |  | 21 |  | 20 |  | 19 |  | 22 |  |  |  |
| Missing | <0.5 |  | <0.5 |  | <0.5 |  | - |  | <0.5 |  |  |  |
| **Parental education** |  |  |  |  |  |  |  |  |  |  |  |  |
| Low | 8 |  | 8 |  | 3 |  | 2 |  | 8 |  | 0.002 |  |
| Medium | 40 |  | 41 |  | 36 |  | 33 |  | 42 |  |  |  |
| High | 46 |  | 51 |  | 60 |  | 65 |  | 50 |  |  |  |
| Missing | 6 |  | <0.5 |  | <0.5 |  | <0.5 |  | <0.5 |  |  |  |
| Income |  |  |  |  |  |  |  |  |  |  |  |  |
| Q1 | 24 |  | 24 |  | 15 |  | 13 |  | 24 |  | 0.011 |  |
| Q2 | 25 |  | 25 |  | 24 |  | 23 |  | 25 |  |  |  |
| Q3 | 25 |  | 25 |  | 29 |  | 30 |  | 25 |  |  |  |
| Q4 | 25 |  | 25 |  | 31 |  | 33 |  | 25 |  |  |  |
| Missing | <0.5 |  | <0.5 |  | <0.5 |  | <0.5 |  | <0.5 |  |  |  |
| **Parental cohabitation** | |  |  |  |  |  |  |  |  |  |  |  |
| Yes | 71 |  | 71 |  | 75 |  | 78 |  | 70 |  | 0.007 |  |
| No / missing ^*^ | 29 |  | 29 |  | 25 |  | 22 |  | 30 |  |  |  |
| **Out of home placement** | |  |  |  |  |  |  |  |  |  |  |  |
| Yes | 1 |  | 1 |  | <0.5 |  | <0.5 |  | 1 |  | 0.012 |  |
| No | 99 |  | 99 |  | 100 |  | 100 |  | 99 |  |  |  |
| **Psychiatric disorder child** | |  |  |  |  |  |  |  |  |  |  |  |
| Yes | 4 |  | 5 |  | 4 |  | 3 |  | 5 |  | 0.005 |  |
| No | 96 |  | 95 |  | 96 |  | 97 |  | 95 |  |  |  |
| **Parental psychiatric disorder** | |  |  |  |  |  |  |  |  |  |  |  |
| Yes | 18 |  | 21 |  | 17 |  | 15 |  | 21 |  | 0.002 |  |
| No | 82 |  | 79 |  | 83 |  | 85 |  | 79 |  |  |  |
| ^*^ Too few observations to report  ^a^ Sex, maternal and paternal age, urbanicity, and parity were measured at birth. Parental education, equivalized household income, parental cohabitation status, out-of-home placement, childhood psychiatric diagnoses, and parental psychiatric diagnoses were assessed up to or at the child's age of 10 years | | | | | | | | | | | |  |
|  | | | | | | | | | | | |  |
|  |  |  |  |  |  |  |  |  |  |  |  |  |

| **Table S6:** Incidence Rate Ratio (IRR) and 95% confidence interval (95% CI) for diagnosed eating disorders (EDs), overall and stratified by sex, across trajectories of childhood adversities in the total population (N=500,240) | | | | | | | | | | | | | |
| --- | --- | --- | --- | --- | --- | --- | --- | --- | --- | --- | --- | --- | --- |
|  |  |  | **Model 1 ^a^** | | |  | **Model 2 ^b^** | | |  | **Model 3 ^c^** | | |
| **All** | **N total** | **N cases** | **IRR** | **95% CI** | |  | **IRR** | **95% CI** | |  | **IRR** | **95% CI** | |
| Low | 361,179 | 3,097 | 1.00 |  |  |  | 1.00 |  |  |  | 1.00 |  |  |
| Material deprivation | 89,756 | 658 | 0.86 | 0.74 | 0.99 |  | 1.02 | 0.88 | 1.18 |  | 0.98 | 0.84 | 1.13 |
| Loss or threat of loss | 27,437 | 259 | 1.10 | 0.88 | 1.37 |  | 1.18 | 0.94 | 1.45 |  | 1.15 | 0.92 | 1.41 |
| High | 21,868 | 201 | 1.07 | 0.83 | 1.37 |  | 1.28 | 0.99 | 1.63 |  | 1.15 | 0.89 | 1.46 |
|  |  |  |  |  |  |  |  |  |  |  |  |  |  |
| **Females** | **N total** | **N cases** | **IRR** | **95% CI** | |  | **IRR** | **95% CI** | |  | **IRR** | **95% CI** | |
| Low | 175,717 | 2,834 | 1.00 |  |  |  | 1.00 |  |  |  | 1.00 |  |  |
| Material deprivation | 43,819 | 588 | 0.84 | 0.71 | 0.97 |  | 1.00 | 0.86 | 1.16 |  | 0.95 | 0.82 | 1.11 |
| Loss or threat of loss | 13,345 | 228 | 1.06 | 0.83 | 1.33 |  | 1.14 | 0.90 | 1.41 |  | 1.10 | 0.88 | 1.37 |
| High | 10,604 | 175 | 1.02 | 0.78 | 1.32 |  | 1.24 | 0.94 | 1.59 |  | 1.10 | 0.85 | 1.42 |
|  |  |  |  |  |  |  |  |  |  |  |  |  |  |
| **Males** | **N total** | **N cases** | **IRR** | **95% CI** | |  | **IRR** | **95% CI** | |  | **IRR** | **95% CI** | |
| Low | 185,462 | 263 | 1.00 |  |  |  | 1.00 |  |  |  | 1.00 |  |  |
| Material deprivation | 45,937 | 70 | 1.08 | 0.61 | 1.81 |  | 1.22 | 0.66 | 2.14 |  | 1.20 | 0.65 | 2.10 |
| Loss or threat of loss | 14,092 | 31 | 1.55 | 0.66 | 3.11 |  | 1.59 | 0.66 | 3.27 |  | 1.57 | 0.64 | 3.25 |
| High | 11,264 | 26 | 1.63 | 0.64 | 3.44 |  | 1.76 | 0.65 | 3.96 |  | 1.70 | 0.63 | 3.75 |
| ^a^ Unadjusted ^b^ Adjusted for maternal and paternal age at birth, parental origin, urbanicity at birth, birth year, and parental education (highest attained or ongoing) at birth ^c^ Adjusted for maternal and paternal age at birth, parental origin, urbanicity at birth, birth year | | | | | | | | | | | | | |

| **Table S7:** Incidence Rate Ratio (IRR) and 95% confidence interval (95% CI) for diagnosed eating disorder subtypes anorexia nervosa (AN), bulimia nervosa (BN) and eating disorders not otherwise specified (EDNOS), across trajectories of childhood adversities in the total population (N=500,240) | | | | | | | | | | | | | |
| --- | --- | --- | --- | --- | --- | --- | --- | --- | --- | --- | --- | --- | --- |
|  |  |  | **Model 1 ^a^** | | |  | **Model 2 ^b^** | | |  | **Model 3 ^c^** | | |
| **AN** | **N total** | **N cases** | **IRR** | **95% CI** | |  | **IRR** | **95% CI** | |  | **IRR** | **95% CI** | |
| Low | 361,179 | 1,978 | 1.00 |  |  |  | 1.00 |  |  |  | 1.00 |  |  |
| Material deprivation | 89,756 | 358 | 0.73 | 0.60 | 0.89 |  | 0.92 | 0.75 | 1.12 |  | 0.86 | 0.70 | 1.05 |
| Loss or threat of loss | 27,437 | 148 | 0.99 | 0.73 | 1.30 |  | 1.07 | 0.79 | 1.42 |  | 1.03 | 0.76 | 1.37 |
| High | 21,868 | 99 | 0.83 | 0.57 | 1.15 |  | 1.11 | 0.76 | 1.56 |  | 0.94 | 0.64 | 1.32 |
|  |  |  |  |  |  |  |  |  |  |  |  |  |  |
| **BN** | **N total** | **N cases** | **IRR** | **95% CI** | |  | **IRR** | **95% CI** | |  | **IRR** | **95% CI** | |
| Low | 361,179 | 364 | 1.00 |  |  |  | 1.00 |  |  |  | 1.00 |  |  |
| Material deprivation | 89,756 | 83 | 0.92 | 0.68 | 1.24 |  | 1.09 | 0.79 | 1.47 |  | 1.04 | 0.76 | 1.40 |
| Loss or threat of loss | 27,437 | 23 | 0.83 | 0.47 | 1.37 |  | 0.89 | 0.50 | 1.46 |  | 0.87 | 0.49 | 1.42 |
| High | 21,868 | 25 | 1.14 | 0.65 | 1.83 |  | 1.36 | 0.77 | 2.22 |  | 1.21 | 0.70 | 1.95 |
|  |  |  |  |  |  |  |  |  |  |  |  |  |  |
| **EDNOS** | **N total** | **N cases** | **IRR** | **95% CI** | |  | **IRR** | **95% CI** | |  | **IRR** | **95% CI** | |
| Low | 361,179 | 1,515 | 1.00 |  |  |  | 1.00 |  |  |  | 1.00 |  |  |
| Material deprivation | 89,756 | 351 | 0.94 | 0.76 | 1.14 |  | 1.07 | 0.88 | 1.30 |  | 1.05 | 0.86 | 1.27 |
| Loss or threat of loss | 27,437 | 146 | 1.27 | 0.93 | 1.69 |  | 1.34 | 1.00 | 1.74 |  | 1.32 | 0.99 | 1.72 |
| High | 21,868 | 132 | 1.44 | 1.04 | 1.94 |  | 1.58 | 1.16 | 2.12 |  | 1.49 | 1.10 | 1.98 |
| ^a^ Unadjusted ^b^ Adjusted for maternal and paternal age at birth, parental origin, urbanicity at birth, birth year, and parental education (highest attained or ongoing) at birth ^c^ Adjusted for maternal and paternal age at birth, parental origin, urbanicity at birth, and birth year | | | | | | | | | | | | | |

| **Table S8:** Incidence Rate Ratio (IRR) and 95% confidence interval (95% CI) for diagnosed eating disorder subtypes anorexia nervosa (AN), bulimia nervosa (BN) and eating disorders not otherwise specified (EDNOS), across trajectories of childhood adversities restricted to females only in the total population (N=243,485) | | | | | | | | | | | | | |
| --- | --- | --- | --- | --- | --- | --- | --- | --- | --- | --- | --- | --- | --- |
|  |  |  | **Model 1 ^a^** | | |  | **Model 2 ^b^** | | |  | **Model 3 ^c^** | | |
| **AN** | **N total** | **N cases** | **IRR** | **95% CI** | |  | **IRR** | **95% CI** | |  | **IRR** | **95% CI** | |
| Low | 175,717 | 1,849 | 1.00 |  |  |  | 1.00 |  |  |  | 1.00 |  |  |
| Material deprivation | 438,19 | 329 | 0.72 | 0.58 | 0.87 |  | 0.91 | 0.73 | 1.12 |  | 0.85 | 0.68 | 1.04 |
| Loss or threat of loss | 13,345 | 134 | 0.96 | 0.70 | 1.28 |  | 1.04 | 0.76 | 1.40 |  | 1.00 | 0.73 | 1.34 |
| High | 10,604 | 90 | 0.81 | 0.55 | 1.14 |  | 1.09 | 0.73 | 1.56 |  | 0.92 | 0.62 | 1.31 |
|  |  |  |  |  |  |  |  |  |  |  |  |  |  |
| **BN** | **N total** | **N cases** | **IRR** | **95% CI** | |  | **IRR** | **95% CI** | |  | **IRR** | **95% CI** | |
| Low | 175,717 | N=482 ^d^ | 1.00 |  |  |  | 1.00 |  |  |  | 1.00 |  |  |
| Material deprivation | 43,819 |  | 0.91 | 0.66 | 1.23 |  | 1.07 | 0.78 | 1.46 |  | 1.02 | 0.74 | 1.38 |
| Loss or threat of loss | 13,345 |  | 0.86 | 0.48 | 1.41 |  | 0.92 | 0.52 | 1.51 |  | 0.89 | 0.50 | 1.46 |
| High | 10,604 |  | 1.17 | 0.67 | 1.89 |  | 1.41 | 0.80 | 2.31 |  | 1.24 | 0.71 | 2.00 |
|  |  |  |  |  |  |  |  |  |  |  |  |  |  |
| **EDNOS** | **N total** | **N cases** | **IRR** | **95% CI** | |  | **IRR** | **95% CI** | |  | **IRR** | **95% CI** | |
| Low | 175,717 | 1,351 | 1.00 |  |  |  | 1.00 |  |  |  | 1.00 |  |  |
| Material deprivation | 43,819 | 300 | 0.89 | 0.72 | 1.10 |  | 1.03 | 0.84 | 1.25 |  | 1.00 | 0.82 | 1.22 |
| Loss or threat of loss | 13,345 | 124 | 1.21 | 0.88 | 1.63 |  | 1.28 | 0.95 | 1.68 |  | 1.26 | 0.94 | 1.65 |
| High | 10,604 | 113 | 1.39 | 0.99 | 1.89 |  | 1.54 | 1.11 | 2.07 |  | 1.44 | 1.05 | 1.92 |
| ^a^ Unadjusted ^b^ Adjusted for maternal and paternal age at birth, parental origin, urbanicity at birth, birth year, and parental education (highest attained or ongoing) at birth ^c^ Adjusted for maternal and paternal age at birth, parental origin, urbanicity at birth, and birth year  ^d^ Total number of cases, as the difference in number of cases from Table S7 is too small to report for each category ^*^ Unweighted RRR (95% CI) | | | | | | | | | | | | | |

| **Table S9**: Odds ratio (OR) and 95% confidence interval (95% CI) for any eating disorders (EDs), including both diagnosed and threshold EDs, and relative risk ratio (RRR) and 95% CI for diagnosed EDs and non-diagnosed EDs across trajectories of childhood adversities in the 18-year follow-up in the Danish National Birth Cohort (DNBC-18; N=43,687) | | | | | | | | | | | | | | | | | | | | | | | | | | |
| --- | --- | --- | --- | --- | --- | --- | --- | --- | --- | --- | --- | --- | --- | --- | --- | --- | --- | --- | --- | --- | --- | --- | --- | --- | --- | --- |
| **Any EDs** | | | | | | | | | | | | | | | | | | | | | | | | | | |
|  |  |  |  | **Model 1 ^a^** | | |  | **Model 2 ^b^** | | |  | **Model 3 ^c^** | | |  | **Model 1 ^a*^** | | |  | **Model 2 ^b*^** | | |  | **Model 3 ^c*^** | | |
|  | **N total** | **N cases** |  | **OR** | **95% CI** | |  | **OR** | **95% CI** | |  | **OR** | **95% CI** | |  | **OR** | **95% CI** | |  | **OR** | **95% CI** | |  | **OR** | **95% CI** | |
| Low | 35,857 | 1,333 |  | 1.00 |  |  |  | 1.00 |  |  |  | 1.00 |  |  |  | 1.00 |  |  |  | 1.00 |  |  |  | 1.00 |  |  |
| Material deprivation | 4,668 | 190 |  | 1.07 | 0.92 | 1.24 |  | 1.05 | 0.90 | 1.22 |  | 1.10 | 0.94 | 1.27 |  | 1.10 | 0.92 | 1.24 |  | 1.06 | 0.90 | 1.23 |  | 1.10 | 0.94 | 1.28 |
| Loss or threat of loss | 2,169 | 79 |  | 0.99 | 0.77 | 1.24 |  | 0.96 | 0.75 | 1.22 |  | 0.99 | 0.77 | 1.25 |  | 0.98 | 0.77 | 1.24 |  | 0.96 | 0.76 | 1.20 |  | 0.98 | 0.77 | 1.22 |
| High | 993 | 58 |  | 1.59 | 1.29 | 1.95 |  | 1.50 | 1.20 | 1.86 |  | 1.67 | 1.34 | 2.05 |  | 1.61 | 1.29 | 1.95 |  | 1.47 | 1.10 | 1.92 |  | 1.60 | 1.21 | 2.09 |
|  |  |  |  |  |  |  |  |  |  |  |  |  |  |  |  |  |  |  |  |  |  |  |  |  |  |  |
| **Diagnosed EDs** | | | | | | | | | | | | | | | | | | | | | | | | | | |
|  | **N total** | **N cases** |  | **RRR** | **95% CI** | |  | **RRR** | **95% CI** | |  | **RRR** | **95% CI** | |  | **RRR** | **95% CI** | |  | **RRR** | **95% CI** | |  | **RRR** | **95% CI** | |
| Low | 35,857 | 378 |  | 1.00 |  |  |  | 1.00 |  |  |  | 1.00 |  |  |  | 1.00 |  |  |  | 1.00 |  |  |  | 1.00 |  |  |
| Material deprivation | 4,668 | 47 |  | 0.95 | 0.71 | 1.27 |  | 1.00 | 0.74 | 1.34 |  | 0.99 | 0.73 | 1.33 |  | 0.96 | 0.71 | 1.3 |  | 0.96 | 0.71 | 1.31 |  | 0.97 | 0.71 | 1.32 |
| Loss or threat of loss | 2,169 | 26 |  | 1.09 | 0.71 | 1.67 |  | 1.12 | 0.73 | 1.72 |  | 1.12 | 0.73 | 1.71 |  | 1.14 | 0.76 | 1.69 |  | 1.15 | 0.77 | 1.71 |  | 1.15 | 0.77 | 1.72 |
| High | 993 | 8 |  | 0.72 | 0.41 | 1.25 |  | 0.80 | 0.45 | 1.42 |  | 0.77 | 0.44 | 1.35 |  | 0.78 | 0.39 | 1.58 |  | 0.8 | 0.39 | 1.62 |  | 0.8 | 0.39 | 1.62 |
|  |  |  |  |  |  |  |  |  |  |  |  |  |  |  |  |  |  |  |  |  |  |  |  |  |  |  |
| **Non-diagnosed EDs** | | | | | | | | | | | | | | | | | | | | | | | | | | |
|  | **N total** | **N cases** |  | **RRR** | **95% CI** | |  | **RRR** | **95% CI** | |  | **RRR** | **95% CI** | |  | **RRR** | **95% CI** | |  | **RRR** | **95% CI** | |  | **RRR** | **95% CI** | |
| Low | 35,857 | 955 |  | 1.00 |  |  |  | 1.00 |  |  |  | 1.00 |  |  |  | 1.00 |  |  |  | 1.00 |  |  |  | 1.00 |  |  |
| Material deprivation | 4,668 | 143 |  | 1.11 | 0.94 | 1.32 |  | 1.07 | 0.89 | 1.27 |  | 1.14 | 0.96 | 1.36 |  | 1.15 | 0.97 | 1.38 |  | 1.09 | 0.91 | 1.31 |  | 1.15 | 0.96 | 1.37 |
| Loss or threat of loss | 2,169 | 53 |  | 0.95 | 0.71 | 1.26 |  | 0.91 | 0.68 | 1.21 |  | 0.94 | 0.71 | 1.26 |  | 0.92 | 0.69 | 1.21 |  | 0.89 | 0.67 | 1.18 |  | 0.91 | 0.69 | 1.2 |
| High | 993 | 50 |  | 1.93 | 1.54 | 2.41 |  | 1.73 | 1.37 | 2.20 |  | 2.01 | 1.6 | 2.53 |  | 1.93 | 1.44 | 2.59 |  | 1.71 | 1.26 | 2.30 |  | 1.91 | 1.42 | 2.56 |
| ^a^ Unadjusted ^b^ Adjusted for maternal and paternal age at birth, urbanicity at birth, birth year, and parental education (highest attained or ongoing) at birth ^c^ Adjusted for maternal and paternal age at birth, urbanicity at birth, and birth year  ^*^ Unweighted OR, RRR (95% CI) | | | | | | | | | | | | | | | | | | | | | | | | | | |
|  | | | | | | | | | | | | | | | | | | | | | | | | | | |

| **Table S10:** Relative Risk Ratio (RRR) and 95% confidence interval (95% CI) for diagnosed eating disorders (ED) and non-diagnosed EDs across trajectories of childhood adversities, restricted to females only in the 18-year follow-up in the Danish National Birth Cohort (DNBC-18; N=25,636) | | | | | | | | | | | | | | | | | | | | | | | | | | |
| --- | --- | --- | --- | --- | --- | --- | --- | --- | --- | --- | --- | --- | --- | --- | --- | --- | --- | --- | --- | --- | --- | --- | --- | --- | --- | --- |
| **Diagnosed EDs** | | | | | | | | | | | | | | | | | | | | | | | | | | |
|  |  |  |  | **Model 1 ^a^** | | |  | **Model 2 ^b^** | | |  | **Model 3 ^c^** | | |  | **Model 1 ^a*^** | | |  | **Model 2 ^b*^** | | |  | **Model 3 ^c*^** | | |
|  | **N total** | **N cases** |  | **RRR** | **95% CI** | |  | **RRR** | **95% CI** | |  | **RRR** | **95% CI** | |  | **RRR** | **95% CI** | |  | **RRR** | **95% CI** | |  | **RRR** | **95% CI** | |
| Low | 20,990 | N=428 ^d^ |  | 1.00 |  |  |  | 1.00 |  |  |  | 1.00 |  |  |  | 1.00 |  |  |  | 1.00 |  |  |  | 1.00 |  |  |
| Material deprivation | 2,763 |  |  | 0.95 | 0.69 | 1.29 |  | 1.02 | 0.74 | 1.40 |  | 0.99 | 0.72 | 1.35 |  | 0.93 | 0.67 | 1.27 |  | 0.94 | 0.68 | 1.30 |  | 0.94 | 0.68 | 1.29 |
| Loss or threat of loss | 1,280 |  |  | 0.96 | 0.59 | 1.56 |  | 1.01 | 0.62 | 1.63 |  | 0.99 | 0.61 | 1.61 |  | 1.01 | 0.66 | 1.56 |  | 1.04 | 0.67 | 1.60 |  | 1.03 | 0.67 | 1.60 |
| High | 603 |  |  | 0.82 | 0.46 | 1.43 |  | 0.98 | 0.55 | 1.76 |  | 0.91 | 0.51 | 1.61 |  | 0.81 | 0.40 | 1.65 |  | 0.87 | 0.43 | 1.79 |  | 0.85 | 0.42 | 1.73 |
|  | | | | | | | | | | | | | | | | | | | | | | | | | | |
| **Non-diagnosed EDs** | | | | | | | | | | | | | | | | | | | | | | | | | | |
|  | **N total** | **N cases** |  | **RRR** | **95% CI** | |  | **RRR** | **95% CI** | |  | **RRR** | **95% CI** | |  | **RRR** | **95% CI** | |  | **RRR** | **95% CI** | |  | **RRR** | **95% CI** | |
| Low | 20,990 | N=1,119 ^d^ |  | 1.00 |  |  |  | 1.00 |  |  |  | 1.00 |  |  |  | 1.00 |  |  |  | 1.00 |  |  |  | 1.00 |  |  |
| Material deprivation | 2,763 |  |  | 1.16 | 0.97 | 1.39 |  | 1.10 | 0.91 | 1.32 |  | 1.18 | 0.98 | 1.41 |  | 1.16 | 0.96 | 1.40 |  | 1.10 | 0.91 | 1.33 |  | 1.15 | 0.95 | 1.39 |
| Loss or threat of loss | 1,280 |  |  | 0.96 | 0.71 | 1.31 |  | 0.94 | 0.69 | 1.27 |  | 0.97 | 0.71 | 1.31 |  | 0.90 | 0.67 | 1.21 |  | 0.88 | 0.66 | 1.18 |  | 0.90 | 0.67 | 1.20 |
| High | 603 |  |  | 2.09 | 1.65 | 2.65 |  | 1.89 | 1.48 | 2.43 |  | 2.18 | 1.71 | 2.77 |  | 1.91 | 1.41 | 2.59 |  | 1.71 | 1.25 | 2.34 |  | 1.90 | 1.40 | 2.59 |
| ^a^ Unadjusted ^b^ Adjusted for maternal and paternal age at birth, urbanicity at birth, birth year, and parental education (highest attained or ongoing) at birth ^c^ Adjusted for maternal and paternal age at birth, urbanicity at birth, and birth year  ^d^ Total number of cases, as the difference in number of cases from Table S9 is too small to report for each category  * Unweighted RRR (95% CI) | | | | | | | | | | | | | | | | | | | | | | | | | | |

| **Table S11:** Incidence Rate Ratio (IRR) and 95% confidence interval (95% CI) for diagnosed eating disorders (ED) and trajectories of childhood adversities across the total population, background population, invited to the 18-year follow-up in the Danish National Birth Cohort (DNBC-18) and DNBC-18 weighted and unweighted. Estimates are presented as unadjusted. | | | | | | | | | | | | | | | | | | | | | | | |
| --- | --- | --- | --- | --- | --- | --- | --- | --- | --- | --- | --- | --- | --- | --- | --- | --- | --- | --- | --- | --- | --- | --- | --- |
|  | **Total population** | | | | | **Background population** | | | | | **Invited DNBC-18** | | | | | **DNBC-18 population** | | | | | **DNBC-18 population weighted** | | |
|  | **N total** | **N cases** | **IRR** | **95% CI** | | **N total** | **N cases** | **IRR** | **95% CI** | | **N total** | **N cases** | **IRR** | **95% CI** | | **N total** | **N cases** | **IRR** | **95% CI** | | **IRR** | **95% CI** | |
| Low | 361,179 | 3,097 | 1.00 |  |  | 238,118 | 2,366 | 1.00 |  |  | 70,474 | 729 | 1.00 |  |  | 35,857 | 378 | 1.00 |  |  | 1.00 |  |  |
| Material deprivation | 89,756 | 658 | 0.86 | 0.74 | 0.99 | 56,793 | 494 | 0.88 | 0.75 | 1.03 | 10,081 | 96 | 0.92 | 0.60 | 1.34 | 4,668 | 47 | 0.95 | 0.58 | 1.49 | 0.95 | 0.58 | 1.46 |
| Loss or threat of loss | 27,437 | 259 | 1.10 | 0.88 | 1.37 | 18,454 | 203 | 1.11 | 0.87 | 1.39 | 4,444 | 49 | 1.07 | 0.59 | 1.76 | 2,169 | 26 | 1.14 | 0.58 | 2.01 | 1.09 | 0.52 | 2.00 |
| High | 21,868 | 201 | 1.07 | 0.83 | 1.37 | 15,095 | 154 | 1.03 | 0.78 | 1.33 | 2,527 | 33 | 1.26 | 0.61 | 2.28 | 993 | 8 | 0.76 | 0.20 | 1.93 | 0.70 | 0.26 | 1.51 |

| **Table S12:** Incidence Rate Ratio (IRR) and 95% confidence interval (95% CI) for diagnosed eating disorders (EDs) across trajectories of childhood adversities in the total population, restricted to no parental EDs before the child’s 10^th^ birthday (N=497,342) | | | | | | | | | | | | | |
| --- | --- | --- | --- | --- | --- | --- | --- | --- | --- | --- | --- | --- | --- |
|  |  |  | **Model 1 ^a^** | | |  | **Model 2 ^b^** | | |  | **Model 3 ^c^** | | |
|  | **N total** | **N cases** | **IRR** | **95% CI** | |  | **IRR** | **95% CI** | |  | **IRR** | **95% CI** | |
| Low | 359,554 | N=4,157 ^d^ | 1.00 |  |  |  | 1.00 |  |  |  | 1.00 |  |  |
| Material deprivation | 89,266 |  | 0.86 | 0.74 | 0.99 |  | 1.02 | 0.87 | 1.18 |  | 0.97 | 0.84 | 1.13 |
| Loss or threat of loss | 27,233 |  | 1.09 | 0.86 | 1.36 |  | 1.16 | 0.92 | 1.43 |  | 1.13 | 0.90 | 1.39 |
| High | 21,289 |  | 1.04 | 0.80 | 1.34 |  | 1.22 | 0.93 | 1.56 |  | 1.10 | 0.84 | 1.40 |
| ^a^ Unadjusted ^b^ Adjusted for maternal and paternal age at birth, parental origin, urbanicity at birth, birth year, and parental education (highest attained or ongoing) at birth ^c^ Adjusted for maternal and paternal age at birth, parental origin, urbanicity at birth, and birth year  ^d^ Total number of cases, as the difference in number of cases from Table S6 was too small to report for each category | | | | | | | | | | | | | |

| **Table S13:** Relative Risk Ratio (RRR) and 95% confidence interval (95% CI) for diagnosed eating disorders (EDs) and non-diagnosed EDs across trajectories of childhood adversities in the total population, restricted to no parental EDs before the child’s 10^th^ birthday (N=43,453) | | | | | | | | | | | | | | | | | | | | | | | | | |
| --- | --- | --- | --- | --- | --- | --- | --- | --- | --- | --- | --- | --- | --- | --- | --- | --- | --- | --- | --- | --- | --- | --- | --- | --- | --- |
| **Diagnosed EDs** | | | | | | | | | | | | | | | | | | | | | | | | | |
|  |  |  | **Model 1 ^a^** | | |  | **Model 2 ^b^** | | |  | **Model 3 ^b^** | | |  | **Model 1 ^a*^** | | |  | **Model 2 ^b*^** | | |  | **Model 3 ^c*^** | | |
|  | **N total** | **N cases** **^e^** | **RRR** | **95% CI** | |  | **RRR** | **95% CI** | |  | **RRR** | **95% CI** | |  | **RRR** | **95% CI** | |  | **RRR** | **95% CI** | |  | **RRR** | **95% CI** | |
|  |  |  |  |  |  |  |  |  |  |  |  |  |  |  |  |  |  |  |  |  |  |  |  |  |  |
| Low | 35,698 | - | 1.00 |  |  |  | 1.00 |  |  |  | 1.00 |  |  |  | 1.00 |  |  |  | 1.00 |  |  |  | 1.00 |  |  |
| Material deprivation | 4,635 |  | 0.94 | 0.70 | 1.27 |  | 0.99 | 0.73 | 1.34 |  | 0.98 | 0.73 | 1.32 |  | 0.94 | 0.69 | 1.29 |  | 0.95 | 0.69 | 1.29 |  | 0.96 | 0.70 | 1.30 |
| Loss or threat of loss | 2,154 |  | 1.10 | 0.71 | 1.69 |  | 1.13 | 0.73 | 1.73 |  | 1.12 | 0.73 | 1.72 |  | 1.14 | 0.77 | 1.71 |  | 1.15 | 0.77 | 1.72 |  | 1.16 | 0.78 | 1.73 |
| High | 966 |  | 0.73 | 0.42 | 1.28 |  | 0.81 | 0.46 | 1.45 |  | 0.79 | 0.45 | 1.38 |  | 0.80 | 0.40 | 1.62 |  | 0.82 | 0.40 | 1.66 |  | 0.82 | 0.40 | 1.66 |
|  |  |  |  |  |  |  |  |  |  |  |  |  |  |  |  |  |  |  |  |  |  |  |  |  |  |
| **Non-diagnosed EDs** | | | | | | | | | | | | | | | | | | | | | | | | | |
|  | **N total** | **N cases ^d^** | **RRR** | **95% CI** | |  | **RRR** | **95% CI** | |  | **RRR** | **95% CI** | |  | **RRR** | **95% CI** | |  | **RRR** | **95% CI** | |  | **RRR** | **95% CI** | |
| Low | 35,698 | N=1,192 | 1.00 |  |  |  | 1.00 |  |  |  | 1.00 |  |  |  | 1.00 |  |  |  | 1.00 |  |  |  | 1.00 |  |  |
| Material deprivation | 4,635 |  | 1.11 | 0.93 | 1.32 |  | 1.06 | 0.89 | 1.27 |  | 1.13 | 0.95 | 1.35 |  | 1.15 | 0.96 | 1.38 |  | 1.09 | 0.91 | 1.31 |  | 1.15 | 0.96 | 1.37 |
| Loss or threat of loss | 2,154 |  | 0.93 | 0.70 | 1.24 |  | 0.89 | 0.67 | 1.19 |  | 0.93 | 0.69 | 1.24 |  | 0.91 | 0.68 | 1.20 |  | 0.88 | 0.66 | 1.17 |  | 0.90 | 0.68 | 1.19 |
| High | 966 |  | 1.83 | 1.46 | 2.31 |  | 1.65 | 1.29 | 2.10 |  | 1.90 | 1.50 | 2.41 |  | 1.87 | 1.38 | 2.52 |  | 1.65 | 1.21 | 2.24 |  | 1.85 | 1.36 | 2.50 |
| ^a^ Unadjusted ^b^ Adjusted for maternal and paternal age at birth, urbanicity at birth, birth year, and parental education (highest attained or ongoing) at birth ^c^ Adjusted for maternal and paternal age at birth, urbanicity at birth, and birth year  ^d^ Total number of cases, as the difference in number of cases from Table S9 was too small to report for each category  ^e^ The difference in total number cases from Table S9 was too small to report  ^*^ Unweighted RRR (95% CI) | | | | | | | | | | | | | | | | | | | | | | | | | |

| **Table S14:** Incidence Risk Ratio (IRR) and 95% confidence interval (95% CI) for diagnosed eating disorders (EDs) across trajectories of childhood adversities stratified by birth year 1996 to 1999 and 2000 to 2003 in the total population (N=500,240) | | | | | | | | | | | | | |
| --- | --- | --- | --- | --- | --- | --- | --- | --- | --- | --- | --- | --- | --- |
|  |  |  | **Model 1 ^a^** | | |  | **Model 2 ^b^** | | |  | **Model 3 ^b^** | | |
| **Birth year 1996-1999** | **N total** | **N cases** | **IRR** | **95% CI** | |  | **IRR** | **95% CI** | |  | **IRR** | **95% CI** | |
| Low | 181,012 | 1,506 | 1.00 |  |  |  | 1.00 |  |  |  | 1.00 |  |  |
| Material deprivation | 48,845 | 361 | 0.89 | 0.72 | 1.10 |  | 1.05 | 0.85 | 1.28 |  | 1.00 | 0.81 | 1.22 |
| Loss or threat of loss | 13,070 | 129 | 1.19 | 0.85 | 1.62 |  | 1.28 | 0.93 | 1.71 |  | 1.24 | 0.90 | 1.67 |
| High | 10,384 | 85 | 0.99 | 0.65 | 1.43 |  | 1.15 | 0.77 | 1.66 |  | 1.04 | 0.69 | 1.48 |
|  |  |  |  |  |  |  |  |  |  |  |  |  |  |
| **Birth year 2000-2003** | **N total** | **N cases** | **IRR** | **95% CI** | |  | **IRR** | **95% CI** | |  | **IRR** | **95% CI** | |
| Low | 180,167 | 1,591 | 1.00 |  |  |  | 1.00 |  |  |  | 1.00 |  |  |
| Material deprivation | 40,911 | 297 | 0.83 | 0.66 | 1.02 |  | 0.99 | 0.79 | 1.23 |  | 0.95 | 0.76 | 1.17 |
| Loss or threat of loss | 14,367 | 130 | 1.03 | 0.74 | 1.38 |  | 1.09 | 0.79 | 1.46 |  | 1.06 | 0.77 | 1.42 |
| High | 11,484 | 116 | 1.15 | 0.81 | 1.56 |  | 1.40 | 0.99 | 1.93 |  | 1.26 | 0.90 | 1.72 |
| ^a^ Unadjusted ^b^ Adjusted for maternal and paternal age at birth, parental origin, urbanicity at birth, birth year, and parental education (highest attained or ongoing) at birth ^c^ Adjusted for maternal and paternal age at birth, parental origin, and urbanicity at birth | | | | | | | | | | | | | |

| **Table S15:** Relative Risk Ratio (RRR) and 95% confidence interval (95% CI) for diagnosed eating disorders (EDs) and non-diagnosed EDs across trajectories of childhood adversities, restricted to information on weight and height (body mass index measurement) measured within the past year in the 18-year follow-up in the Danish National Birth Cohort (DNBC-18; N=38,146) | | | | | | | | | | | | | | | | | | | | | | | | | | |
| --- | --- | --- | --- | --- | --- | --- | --- | --- | --- | --- | --- | --- | --- | --- | --- | --- | --- | --- | --- | --- | --- | --- | --- | --- | --- | --- |
| **Diagnosed EDs** | | | | | | | | | | | | | | | | | | | | | | | | | | |
|  |  |  |  | **Model 1 ^a^** | | |  | **Model 2 ^b^** | | |  | **Model 3 ^b^** | | |  | **Model 1 ^a*^** | | |  | **Model 2 ^b*^** | | |  | **Model 3 ^c*^** | | |
|  | **N total** | **N cases ^d^** |  | **RRR** | **95% CI** | |  | **RRR** | **95% CI** | |  | **RRR** | **95% CI** | |  | **RRR** | **95% CI** | |  | **RRR** | **95% CI** | |  | **RRR** | **95% CI** | |
| Low | 31,451 | N=398 |  | 1.00 |  |  |  | 1.00 |  |  |  | 1.00 |  |  |  | 1.00 |  |  |  | 1.00 |  |  |  | 1.00 |  |  |
| Material deprivation | 3,978 |  |  | 1.07 | 0.78 | 1.45 |  | 1.09 | 0.80 | 1.49 |  | 1.10 | 0.80 | 1.50 |  | 1.05 | 0.76 | 1.45 |  | 1.04 | 0.75 | 1.43 |  | 1.05 | 0.76 | 1.45 |
| Loss or threat of loss | 1,890 |  |  | 1.29 | 0.84 | 1.99 |  | 1.32 | 0.85 | 2.03 |  | 1.32 | 0.86 | 2.04 |  | 1.33 | 0.89 | 1.99 |  | 1.34 | 0.89 | 2.00 |  | 1.35 | 0.90 | 2.02 |
| High | 827 |  |  | 0.60 | 0.30 | 1.17 |  | 0.62 | 0.31 | 1.24 |  | 0.62 | 0.31 | 1.22 |  | 0.48 | 0.18 | 1.28 |  | 0.46 | 0.17 | 1.26 |  | 0.48 | 0.18 | 1.28 |
| **Non-diagnosed EDs** | | | | | | | | | | | | | | | | | | | | | | | | | | |
|  | **N total** | **N cases** |  | **RRR** | **95% CI** | |  | **RRR** | **95% CI** | |  | **RRR** | **95% CI** | |  | **RRR** | **95% CI** | |  | **RRR** | **95% CI** | |  | **RRR** | **95% CI** | |
| Low | 31,451 | 808 |  | 1.00 |  |  |  | 1.00 |  |  |  | 1.00 |  |  |  | 1.00 |  |  |  | 1.00 |  |  |  | 1.00 |  |  |
| Material deprivation | 3,978 | 119 |  | 1.12 | 0.93 | 1.35 |  | 1.05 | 0.86 | 1.28 |  | 1.12 | 0.93 | 1.36 |  | 1.17 | 0.96 | 1.42 |  | 1.10 | 0.90 | 1.34 |  | 1.15 | 0.95 | 1.41 |
| Loss or threat of loss | 1,890 | 43 |  | 0.90 | 0.66 | 1.24 |  | 0.86 | 0.62 | 1.19 |  | 0.89 | 0.65 | 1.23 |  | 0.89 | 0.65 | 1.21 |  | 0.86 | 0.63 | 1.17 |  | 0.88 | 0.64 | 1.20 |
| High | 827 | 40 |  | 1.77 | 1.37 | 2.29 |  | 1.50 | 1.14 | 1.97 |  | 1.77 | 1.36 | 2.31 |  | 1.92 | 1.38 | 2.65 |  | 1.64 | 1.17 | 2.30 |  | 1.86 | 1.34 | 2.59 |
| ^a^ Unadjusted ^b^ Adjusted for maternal and paternal age at birth, urbanicity at birth, birth year, and parental education (highest attained or ongoing) at birth ^c^ Adjusted for maternal and paternal age at birth, urbanicity at birth, and birth year  ^d^ Total number of cases, as the difference in number of cases from Table S9 was too small to report for each category ^*^ Unweighted RRR (95% CI) | | | | | | | | | | | | | | | | | | | | | | | | | | |

| **Table S16:** Relative Risk Ratio (RRR) and 95% confidence interval (95% CI) for diagnosed eating disorders (EDs) and non-diagnosed EDs across trajectories of childhood adversities, restricted to only threshold anorexia nervosa (AN) and bulimia nervosa (BN) cases in the 18-year follow-up in the Danish National Birth Cohort (DNBC-18; N=43,137) | | | | | | | | | | | | | | | | | | | | | | | | | | |
| --- | --- | --- | --- | --- | --- | --- | --- | --- | --- | --- | --- | --- | --- | --- | --- | --- | --- | --- | --- | --- | --- | --- | --- | --- | --- | --- |
| **Diagnosed EDs** | | | | | | | | | | | | | | | | | | | | | | | | | | |
|  |  |  |  | **Model 1 ^a^** | | |  | **Model 2 ^b^** | | |  | **Model 3 ^c^** | | |  | **Model 1 ^a*^** | | |  | **Model 2 ^b*^** | | |  | **Model 3 ^c*^** | | |
|  | **N total** | **N cases** |  | **RRR** | **95% CI** | |  | **RRR** | **95% CI** | |  | **RRR** | **95% CI** | |  | **RRR** | **95% CI** | |  | **RRR** | **95% CI** | |  | **RRR** | **95% CI** | |
| Low | 35,424 | 378 |  | 1.00 |  |  |  | 1.00 |  |  |  | 1.00 |  |  |  | 1.00 |  |  |  | 1.00 |  |  |  | 1.00 |  |  |
| Material deprivation | 4,599 | 47 |  | 0.95 | 0.71 | 1.27 |  | 1.00 | 0.74 | 1.34 |  | 0.99 | 0.73 | 1.33 |  | 0.96 | 0.71 | 1.30 |  | 0.96 | 0.71 | 1.31 |  | 0.97 | 0.71 | 1.32 |
| Loss or threat of loss | 2,147 | 26 |  | 1.09 | 0.71 | 1.67 |  | 1.12 | 0.73 | 1.73 |  | 1.12 | 0.73 | 1.71 |  | 1.14 | 0.76 | 1.70 |  | 1.15 | 0.77 | 1.71 |  | 1.15 | 0.77 | 1.72 |
| High | 967 | 8 |  | 0.72 | 0.41 | 1.25 |  | 0.80 | 0.45 | 1.42 |  | 0.77 | 0.44 | 1.35 |  | 0.78 | 0.39 | 1.58 |  | 0.80 | 0.39 | 1.62 |  | 0.80 | 0.39 | 1.62 |
|  |  |  |  |  |  |  |  |  |  |  |  |  |  |  |  |  |  |  |  |  |  |  |  |  |  |  |
| **Non-diagnosed EDs** | | | | | | | | | | | | | | | | | | | | | | | | | | |
|  | **N total** | **N cases** |  | **RRR** | **95% CI** | |  | **RRR** | **95% CI** | |  | **RRR** | **95% CI** | |  | **RRR** | **95% CI** | |  | **RRR** | **95% CI** | |  | **RRR** | **95% CI** | |
| Low | 35,424 | 522 |  | 1.00 |  |  |  | 1.00 |  |  |  | 1.00 |  |  |  | 1.00 |  |  |  | 1.00 |  |  |  | 1.00 |  |  |
| Material deprivation | 4,599 | 74 |  | 1.06 | 0.83 | 1.35 |  | 1.10 | 0.86 | 1.41 |  | 1.12 | 0.88 | 1.43 |  | 1.09 | 0.86 | 1.40 |  | 1.09 | 0.85 | 1.40 |  | 1.11 | 0.87 | 1.43 |
| Loss or threat of loss | 2,147 | 31 |  | 1.10 | 0.76 | 1.59 |  | 1.09 | 0.76 | 1.58 |  | 1.10 | 0.76 | 1.59 |  | 0.98 | 0.68 | 1.41 |  | 0.96 | 0.67 | 1.39 |  | 0.97 | 0.68 | 1.40 |
| High | 967 | 24 |  | 1.62 | 1.16 | 2.25 |  | 1.75 | 1.24 | 2.47 |  | 1.80 | 1.29 | 2.52 |  | 1.70 | 1.12 | 2.57 |  | 1.67 | 1.10 | 2.55 |  | 1.74 | 1.15 | 2.65 |
| ^a^ Unadjusted ^b^ Adjusted for maternal and paternal age at birth, urbanicity at birth, birth year, and parental education (highest attained or ongoing) at birth ^c^ Adjusted for maternal and paternal age at birth, urbanicity at birth, and birth year ^*^ Unweighted RRR (95% CI) | | | | | | | | | | | | | | | | | | | | | | | | | | |

# **Figure S1:** Figure depicting co-occurrence of diagnosed eating disorders (EDs) and threshold EDs, including subtypes of threshold anorexia nervosa (AN), bulimia nervosa (BN), and binge eating disorder (BED) and diagnosed AN, BN, and eating disorder not otherwise specified (EDNOS) in the 18-year follow-up in the Danish National Birth Cohort (DNBC-18; N=43,687)


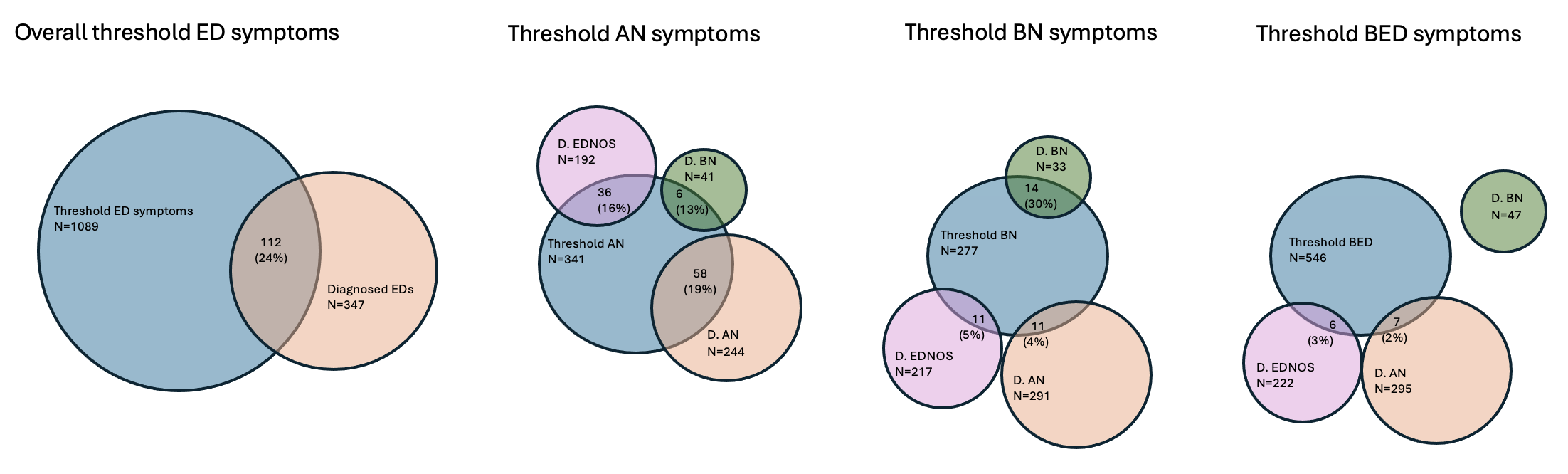
D. = diagnosed
